# Reliability and Validity of a Dog Personality and Unwanted Behavior Survey

**DOI:** 10.3390/ani11051234

**Published:** 2021-04-24

**Authors:** Milla Salonen, Salla Mikkola, Emma Hakanen, Sini Sulkama, Jenni Puurunen, Hannes Lohi

**Affiliations:** 1 Department of Veterinary Biosciences, University of Helsinki, 00014 Helsinki, Finland; milla.ahola@helsinki.fi (M.S.); salla.mikkola@helsinki.fi (S.M.); emma.hakanen@helsinki.fi (E.H.); sini.sulkama@helsinki.fi (S.S.); jenni.puurunen@petbiomics.com (J.P.); 2 Department of Medical and Clinical Genetics, University of Helsinki, 00014 Helsinki, Finland; 3 Folkhälsan Research Center, 00290 Helsinki, Finland

**Keywords:** dog personality, unwanted behavior, behavior problems, behavior assessment, test-retest reliability, inter-rater reliability, convergent validity, discriminant validity, personality structure, survey study

## Abstract

**Simple Summary:**

Dogs have distinct personalities, meaning differences between individuals that persist throughout their lives. However, it is still unclear what traits are required to define the whole personality of dogs. Personality and unwanted behavior are often studied using behavioral questionnaires, but researchers should ensure that these questionnaires are reliable and valid, meaning that they measure the behavior traits they were intended to measure. In this study, we first examined what traits define a dog’s personality. We discovered seven personality traits: Insecurity, Training focus, Energy, Aggressiveness/dominance, Human sociability, Dog sociability and Perseverance. We also studied six unwanted behavior traits: noise sensitivity, fearfulness, aggression (including barking, stranger directed aggression, owner directed aggression and dog directed aggression), fear of surfaces and heights, separation anxiety, and impulsivity/inattention (including hyperactivity/impulsivity and inattention). We examined the reliability of these traits by asking some dog owners to answer to the questionnaire twice, several weeks apart, and by asking another family member to answer the questionnaire of the same dog. Furthermore, we studied the validity of these traits by forming predictions based on previous literature. Based on our results, this personality and unwanted behavior questionnaire is a good tool to study dog behavior.

**Abstract:**

Dogs have distinct, consistent personalities, but the structure of dog personality is still unclear. Dog personality and unwanted behavior are often studied with behavioral questionnaires. Even though many questionnaires are reliable and valid measures of behavior, all new questionnaire tools should be extensively validated. Here, we examined the structure of personality and six unwanted behavior questionnaire sections: noise sensitivity, fearfulness, aggression, fear of surfaces and heights, separation anxiety and impulsivity/inattention with factor analyses. Personality consisted of seven factors: Insecurity, Training focus, Energy, Aggressiveness/dominance, Human sociability, Dog sociability and Perseverance. Most unwanted behavior sections included only one factor, but the impulsivity/inattention section divided into two factors (Hyperactivity/impulsivity and Inattention) and the aggression section into four factors (Barking, Stranger directed aggression, Owner directed aggression and Dog directed aggression). We also examined the internal consistency, test-retest reliability, inter-rater reliability and convergent validity of the 17 personality and unwanted behavior traits and discovered excellent reliability and validity. Finally, we investigated the discriminant validity of the personality traits, which was good. Our findings indicate that this personality and unwanted behavior questionnaire is a reliable and valid tool that can be used to study personality and behavior extensively.

## 1. Introduction

Animal personality traits are behavior traits that are relatively stable over time and across contexts [1,2,3]. Especially in humans and captive animals, the concept of personality often encompasses the combination of traits needed to define an individual’s personality and distinguish individuals from each other [4,5]. Thus, the structure of personality in different animal species interests researchers.

It is still unclear what traits form personality in dogs, as different studies have discovered a different number of traits [6]. For example, the well-validated Dog Personality Questionnaire (DPQ) consists of five personality factors: Fearfulness, Aggression towards people, Aggression towards animals, Activity/excitability, and Responsiveness to training [7]. Similarly well-validated Revised Monash Canine Personality Questionnaire (MCPQ-R) includes five factors: Extraversion, Motivation, Training focus, Amicability, and Neuroticism [8]. Other studies have discovered, for example, four [9,10], five [11,12] or eleven [13] factors. Jones and Gosling [6] categorized traits measured in different studies to six wider domains: activity, which mostly involves motor activity; aggression, including both human directed and dog directed displays of aggressive behavior; sociability, including social behaviors towards both humans and other dogs; responsiveness to training, which includes the tendency to stay focused, willingness to work with people and quickness of learning; submissiveness, the opposite of dominance; and fearfulness, also involving reactivity.

Questionnaires are a commonly used method of collecting behavioral data from companion animals. Despite their frequent use, they are somewhat subjective, as they do not collect data straight from the animals, but their owners. Many surveys, however, are reliable [14,15,16,17,18,19]. Still, new surveys and previously validated surveys that are translated or edited should be extensively validated to ensure that they measure what they are designed to measure.

Nearly a decade ago we designed and validated a behavior questionnaire focused on fearfulness in pet dogs [14]. This was later expanded to include aggressive behavior [20] and a previously used compulsive behavior section [21] and later included sections about fear of surfaces and heights, separation anxiety [22] and impulsive and inattentive behavior adapted from Vas et al. [23].

As we examined the environmental factors associated with behavior [24,25,26,27], it became evident that the survey could use another revision to allow for analysis of variation in behavior traits instead of only case-control studies. Therefore, we redesigned most sections of the questionnaire to utilize factor analysis for forming the scores for each dog. Here, we report the factor structure, reliability and validity of these redesigned survey sections.

## 2. Methods

### 2.1. Questionnaire

The questionnaire included nine behavioral sections: personality, noise sensitivity, fearfulness, separation-related behavior, fear of surfaces and heights, aggression, impulsivity/inattention, cognition, and compulsive behavior. Additionally, the questionnaire included an extensive background section as well as a health section. The questionnaire can be found in Supplementary File: Questionnaire. Here, we report results from other behavior sections except for compulsive behavior, as compulsions are largely separate traits, making scale construction questionable, and cognition, which is based on a validated survey which does not utilize factor analysis [28].

#### 2.1.1. Personality Questionnaire

We developed an adjective-based dog personality questionnaire using a combination of top-down and bottom-up approaches. Firstly, we searched for and utilized adjective-based personality questionnaires designed to be answered by caretakers of different pets and captive animals [8,15,29,30,31,32,33,34,35,36,37]. From these questionnaires, we selected behaviors that were applicable to dogs. After this top-down approach, we added adjectives relevant for dogs particularly. This combined bottom-up/top-down approach allows the inclusion of species-specific behaviors and simultaneously enables comparison between species [35].

After excluding irrelevant, completely overlapping or ambiguous adjectives, the questionnaire included 63 adjectives and definitions (Supplementary File: Questionnaire) in a randomized order. Owners were asked to indicate how strongly they agreed with the answering options being “strongly disagree”, “somewhat disagree”, “neither agree or disagree”, “somewhat agree”, “strongly agree” and “I don’t know”.

#### 2.1.2. Unwanted Behavior Questionnaires

Other behavioral sections focused on possibly unwanted or problematic behaviors, including noise sensitivity (fear of thunder, fireworks, gunshots and other noises), fearfulness (towards unfamiliar people, dogs and situations), separation-related behavior (both when the owner is leaving and when the dog is alone), fear of surfaces and heights, aggression (towards strangers, the owner and other dogs), impulsivity/inattention and compulsive behavior (including, for example, tail chasing, pacing, flank sucking and light/shadow chasing).

The impulsivity/inattention section was developed by Vas and colleagues [23] and translated to Finnish. Other sections were based on our previous questionnaire [22]. We redesigned some sections, and the new questionnaire can be found in Supplementary File: Questionnaire.

### 2.2. Subjects

**Figure 1:**
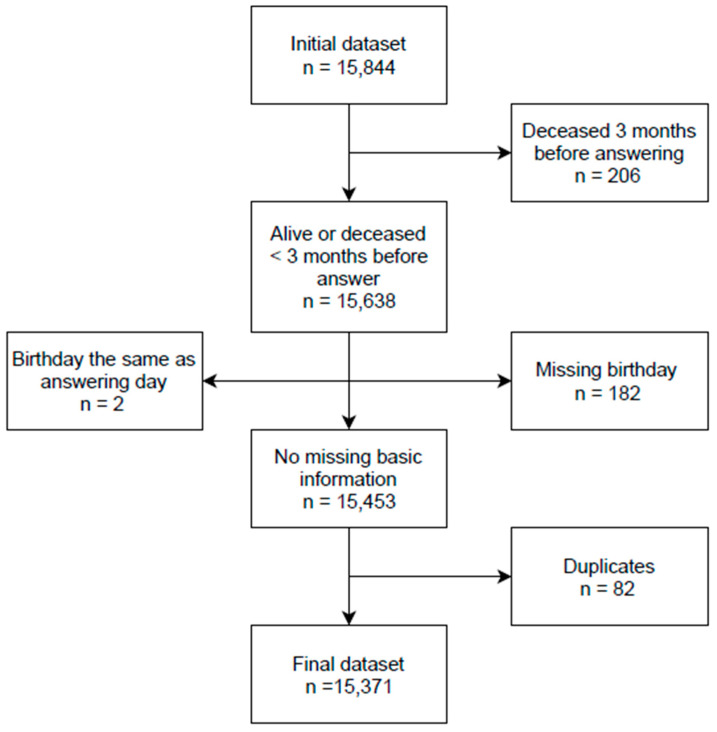
Flow chart of the study population and sample size for dog personality and behavior studies.

Before analyses, we excluded dogs with missing basic information and duplicate answers (Figure 1). Firstly, we excluded dogs that were deceased more than 3 months before answering (206 dogs). We also excluded dogs whose birthdays were not reported by their owners and could not be verified from other sources (182 dogs) and dogs whose birthday was reported to be the date of answering (2 dogs). Some owners did not report their dog’s sex. For these, we tried to verify their sex from other sources and when we could not, we classified the dogs as male/female based on their calling names (39 dogs). Of these, purebred dogs were assumed intact and mixed breed dogs neutered, as this was the case with most dogs in our study population. Finally, some owners had answered for their dogs more than once. From these, we selected the most complete or newest answer.

The final dataset included responses from 15,371 dogs in 329 breeds and breed variants. As many breeds were represented by only a few individuals, we grouped many of them based on the genetic relatedness [38], the purpose of the breed and known similarities in behavior. As a result of this grouping, the final sample included 19 individual breeds, 32 breed groups and mixed breed dogs (Table S1).

During the last months of the data collection, we selected a set of owners who had answered all or most questionnaire sections 1–3 months prior and sent them a request to participate in the test-retest reliability study. These owners were requested to answer the questionnaire sections again.

Finally, we selected a set of owners who had answered all or most questionnaire sections 1–3 months prior and reported to live with another adult. We requested that they participate in the inter-rater reliability study by allowing the other adult family member to answer the questionnaire sections. For this set of questionnaires, we included a question asking how long the other respondent has known the dog. The answering options were “less than 3 months”, “3–6 months”, “6 months to 1 year”, “1–5 years” and “over 5 years”. The participants also had to declare that they had not discussed their dog’s behavior with the other owner when filling the questionnaires.

### 2.3. Convergent and Discriminant Validity

We evaluated the convergent validity of the questionnaire by hypothesis testing (concurrent validity). We collected hypotheses from previous literature and included 1–4 hypotheses per factor (Table S2). For example, based on previous literature, we hypothesized that female dogs would, on average, be more fearful (towards strangers, dogs and situations) than male dogs [9,39,40,41,42,43]. We also hypothesized that dogs whose owners feel that their dog shows a particular unwanted behavior, for example, aggression towards strangers or fear of thunder, would, on average, have higher factor scores than dogs that do not show a particular behavior (Table S2).

For discriminant validity, we evaluated the correlations between factors to see whether factors that should not be correlated. Our previous study indicated that all the unwanted behavior traits we studied are correlated [22] and therefore, we only evaluated the discriminant validity of the personality section.

### 2.4. Statistical Analyses

#### 2.4.1. Factor Analyses

We performed factor analysis for all questionnaire sections to reduce the questionnaire items into a smaller number of biologically meaningful traits. We excluded questions with more than 20% missing responses and dogs with more than 20% missing responses in the remaining items. Before factor analyses, we tested the suitability of our datasets for factor analysis with the Kaiser-Meyer-Olkin test for sampling adequacy from the package psych [44].

We conducted the factor analyses with the package psych [44]. We used polychoric correlation matrices (as all questionnaire items were coded on a Likert scale) and conducted the factor analyses without rotation and with mean imputation. We evaluated the number of factors to be extracted with the scree test and Velicer’s minimum average partial (MAP) test. Furthermore, we evaluated the quality of the factor structure by extracting all possible structures (Goldberg’s hierarchical tree) starting from 1 factor up to at least two factors more than recommended by the scree test. We evaluated the conceptual interpretability of the competing factor structures, as well as compared the root mean square error of approximation (RMSEA) and the Tucker-Lewis index between these structures.

#### 2.4.2. Internal Consistency, Test-Retest Reliability and Inter-Rater Reliability

We calculated Cronbach’s Alpha and Guttman’s Lambda 6 with the package psych [44] for all factors. For test-retest reliability, we used the package psych [44] to calculate the correlations between the first and second time of answering. These correlations were calculated for all items and extracted factors. Finally, for inter-rater reliability, we used the package psych [44] to estimate the inter-rater reliabilities of factors and items based on intraclass correlation coefficients.

#### 2.4.3. Convergent and Discriminant Validity

Before validity analyses, the items that did not load onto any factor (all loadings <0.3) and items that were unreliable based on test-retest and inter-rater reliabilities were removed and the factor analyses were conducted again for the reduced set of items. We then extracted the factor scores for all individual dogs with the package psych [44], using the “tenBerge” estimation method for multifactorial structures and “Thurstone” for unifactorial structures. These factor scores were used in subsequent validity analyses.

To calculate the validity coefficients, we used Pearson correlations for continuous predictors (for example, dog’s age) and Welch *t*-tests for discrete predictors (for example, dog’s sex). We corrected all *p*-values for false discovery rate (FDR) to decrease the probability of type I error. The significance cut-off *p*-value was set at *p* < 0.05.

## 3. Results

### 3.1. Descriptive Statistics

In total, the final dataset included 15,371 dog individuals from 11,498 owners. This sample included dogs in 329 breeds and breed variants, which were grouped to form 31 breed groups and 21 individual breeds (Table S1). Of the dogs, 52.9% were females and 47.1% males. In total, 23.5% of dogs (26.0% of females and 20.7% of males) were neutered. Age varied between 0.16 (2 months) and 18.1 years, with a mean age of 5.23 years (sd 3.47). The number of dogs varied between questionnaire sections (Table 1). In the test-retest reliability and inter-rater reliability datasets, the number of dogs and time between the two answers also varied between sections (Table 1).

### 3.2. Factor Structure

Personality questionnaire items formed seven factors, which were named Insecurity, Training focus, Energy, Aggressiveness/dominance, Human sociability, Dog sociability and Perseverance (Table 2). This factor structure accounted for 54% of the variance in behavior.

Noise sensitivity, fearfulness, fear of surfaces and heights and separation anxiety sections each formed one factor, which included all or most of the items in the particular section. These factors were correspondingly named Noise sensitivity, Fearfulness, Fear of surfaces and heights and Separation anxiety (Tables S3–S6) and accounted for 51%, 34%, 60% and 51% of the variance, respectively. The impulsivity section, in which the items were translated from [23], constituted two factors, as in the original study: Inattention and Hyperactivity/impulsivity (Table S7), which explained 52% of the variance. Aggression section items formed four factors, which were named Barking, Stranger directed aggression, Owner directed aggression and Dog directed aggression (Table S8) and accounted for 63% of the variance.

### 3.3. Reliability and Internal Consistency

Internal consistency of most factors was adequate (Table 3). In the personality questionnaire, Cronbach’s alpha varied from 0.61 (Perseverance) to 0.89 (Insecurity) and Guttmann’s lambda 6 from 0.67 (Perseverance) to 0.91 (Insecurity). In other questionnaire sections, Cronbach’s alpha varied from 0.63 (Owner directed aggression) to 0.95 (Noise sensitivity), with a mean of 0.78. Guttman’s lambda 6 varied from 0.68 (Owner directed aggression) to 0.97 (Noise sensitivity), with a mean of 0.81. Besides personality traits Human sociability and Perseverance, and other traits Owner directed aggression and Dog directed aggression, all estimates were over 0.70.

Test-retest reliability of all factors was good (Table 3). In the personality questionnaire, the correlation between the two timepoints varied from 0.70 (Perseverance) to 0.91 (Insecurity, Training focus and Aggressiveness/dominance). In other sections, the correlation varied from 0.77 (Hyperactivity/impulsivity) to 0.93 (Noise sensitivity). Mean test-retest reliability of all factors was 0.84. Test-retest reliability estimates of individual items can be found in Tables S3–S9.

Inter-rater reliabilities of all factors were similarly good (Table 3). In the personality questionnaire, highest inter-rater reliability was obtained by the Aggressiveness/dominance factor (ICC(1,1) = 0.81, ICC(1,*k*) = 0.90) and the lowest by the Human sociability factor (ICC(1,1) = 0.48, ICC(1,*k*) = 0.65). In other sections, the highest inter-rater reliability was achieved by the Noise sensitivity factor (ICC(1,1) = 0.80, ICC(1,*k*) = 0.89) and the lowest by the Separation anxiety factor (ICC(1,1) = 0.50, ICC(1,*k*) = 0.66). Mean ICC(1,1) of all factors was 0.69 and mean ICC(1,*k*) was 0.81. Inter-rater reliability estimates of individual items can be found in Tables S3–S9.

### 3.4. Convergent and Discriminant Validity

In total, we formed 51 hypotheses to validate 17 factors (Table 4 and Table S2). Of these 51 hypotheses, 47 were met and only 4 did not hold true. These four hypotheses were in Human sociability, Separation anxiety, Barking and Owner directed aggression.

We assessed the discriminant validity of the personality section by evaluating the factor correlations between the factors (Table 5). Moderate correlations (>0.3) were observed only between Training focus and Insecurity and between Aggressiveness/dominance and Dog sociability.

## 4. Discussion

We developed a dog personality and unwanted behavior questionnaire and examined the reliability and validity of the survey sections. Based on our analyses, the survey has excellent reliability and validity. Thus, it should be a reliable and objective method of collecting behavioral data from dog owners.

The dog personality structure we obtained from our questionnaire included seven traits: Insecurity, Training focus, Aggressiveness/dominance, Energy, Dog sociability, Human sociability and Perseverance. Previous studies have not discovered this exact number of personality factors, but, nevertheless, this structure showed similarities to previous dog personality structures. Insecurity factor resembled previous traits labeled Fearfulness [7], Neuroticism [8], Boldness [9] and Curiosity/fearlessness [12]. Training focus factor similarly resembled traits previously named Responsiveness to training [7], Training focus [8], Sociability-obedience [11] and Trainability [9,10,13]. Energy factor was very similar to previous factors called Activity/excitability [7], Extraversion [8], Activity-independence [11], Activity [10] and combined C-BARQ Excitability and Energy factors [13,45]. Aggressiveness/dominance paralleled DPQ Aggression towards animals trait [7], C-BARQ Dog rivalry [45], negative aspects of Kubinyi and others Dog sociability [9] and some aspects of Mirkó and others Aggressiveness [10]. Dog sociability factor resembled Kubinyi and others factor named similarly [9], and shared some similarities with MCPQ-R Amicability trait [8]. Human sociability factor likewise resembled the MCPQ-R Amicability trait [8]. Human sociability traits differed from Svartberg and Forkman Sociability [12] and Mirkó and others Stranger directed sociability [10], as we did not examine sociability with strange people. Finally, perseverance shared similarities with MCPQ-R Motivation [8]. In total, we managed to target all six personality domains suggested by Jones and Gosling [6]: activity (Energy), aggression (Aggressiveness/dominance), sociability (Dog sociability and Human sociability), responsiveness to training (Training focus), submissiveness (Aggressiveness/dominance) and fearfulness (Insecurity). Furthermore, we discovered an additional personality trait, Perseverance.

Of the other questionnaire sections, noise sensitivity, fearfulness, separation anxiety, and fear of surfaces/heights each formed only one factor. In noise sensitivity, this is not surprising, as noise sensitive dogs often display fearfulness towards many different noises [20,22,46,47,48]. In contrast, unexpectedly, fearfulness also comprised only one trait, despite it is often divided into social and non-social fear [49] or fear towards specific targets [13,45]. Therefore, it seems that at least in our dataset and with our questions, fear towards both unfamiliar people, dogs and situations are highly correlated.

The impulsivity/inattention section was divided into two factors and the aggression section into four factors. The factors of the impulsivity/inattention section were Inattention and Hyperactivity/impulsivity, which was also discovered by Vas et al. [23] and by our previous study using their questionnaire (but translated to Finnish) [22]. The factors of the aggression section were Barking, Stranger directed aggression, Owner directed aggression and Dog directed aggression. Another, nearly equally good factor structure split the items into two factors: Meeting aggression (including items related to barking, stranger directed aggression and aggression towards unfamiliar dogs) and Resource/handling aggression (including items related to owner directed aggression and resource aggression towards familiar dogs). We opted to use this four-factor structure as we felt that Barking might be more related to fearfulness. Indeed, despite that Barking was highly correlated with Stranger directed aggression, the correlation between Barking and Fearfulness was much higher than the correlation between Stranger directed aggression and Fearfulness (0.57 and 0.39, respectively). Some previous studies have also indicated that aggression traits are separate [22,50], supported by our results.

The internal consistency of all factors was adequate. In most factors, Cronbach’s alpha exceeded the suggested [51] cut-off of 0.70. Four factors, Human sociability, Perseverance, Owner directed aggression and Dog directed aggression achieved values less than 0.70. Cronbach’s alpha values ranged from 0.61 (Perseverance) to 0.95 (Noise sensitivity), with a mean of 0.78. These Cronbach’s alpha values were similar to previous studies, in which Cronbach’s alpha has varied from less than 0.60 to more than 0.90 [7,8,9,11,12,13,37] and estimates falling between 0.60 and 0.70 are common [9,11,12,13]. Cronbach’s alpha is highly dependent on the length of the scale [7]. Perseverance and Dog directed aggression included only four items and Owner directed aggression only five items, explaining this low internal consistency. Human sociability, on the other hand, included eight items but the loadings of many items were low. Therefore, these scales could be improved by including additional questions that resemble high loading items.

Our personality and unwanted behavior factors had good test-retest reliability. Test-retest reliability, measured by Pearson correlation coefficient, ranged from 0.70 (Perseverance) to 0.93 (Noise sensitivity), with a mean of 0.84. Jones [7] reported even higher test-retest reliability estimates, ranging from 0.88 to 0.94 with a similar retest interval than our study. However, their extensive literature search discovered that the mean test-retest reliability estimate for dog behavior was 0.63 [7].

The inter-rater reliability of our extracted factors was good, with ICC(1,1) values ranging from 0.48 (Human sociability) to 0.81 (Aggressiveness/dominance) and ICC(1,*k*) values ranging from 0.65 (Human sociability) to 0.90 (Aggressiveness/dominance). The mean ICC(1,1) of factors was 0.69 and the mean ICC(1,*k*) was 0.81. In previous studies, ICC values have ranged between 0.07 and 0.98 [7,11,52,53,54], with most values varying between 0.50 and 0.80. In human and animal personality studies, the mean interobserver agreement is around 0.50 [55]. For example, in one study, ICC values of human five-factor model subscales varied between 0.30 and 0.65 [56]. In human personality research, Cicchetti [51] suggested having ICC values of at least 0.4 but preferably over 0.6. Thus, our inter-rater reliability estimates are mostly excellent and in line with previous studies.

To examine the convergent validity of our questionnaire, we formed 51 hypotheses primarily based on previous literature. Of these 51 hypotheses, only four were not met. Based on one study [8], we hypothesized that large dogs would have a higher mean Human sociability than small dogs but instead discovered the opposite association, even though the Pearson correlation coefficient was small (−0.09). Unlike we hypothesized, large dogs did not display less separation anxiety [41,57,58], as separation anxiety was not associated with body size. Furthermore, we hypothesized that male dogs would bark more [42] and that older dogs would show more owner directed aggression [59,60], but these factors were not associated with these behaviors. Other hypotheses held true, and all of these four traits also included hypotheses that were met. Furthermore, in all unwanted behavior sections, we hypothesized that dogs who were reported to display the particular trait would show a higher mean score than dogs reported not to display the behavior. Indeed, the difference in means between these groups was large and highly significant.

Finally, we examined the discriminant validity of our personality questionnaire by examining the correlations between personality factors. Moderate (Pearson correlation coefficient more than 0.30) were observed only between Dog sociability and Aggressiveness/dominance, and between Insecurity and Training focus. This correlation between Dog sociability and Aggressiveness/dominance is not surprising, as Aggressiveness/dominance was comprised of items related to behavior towards other dogs and as in one previous study, similar items have loaded onto a single factor [9]. The correlation between Insecurity and Training focus is, however, interesting, as it has not been observed previously. Training focus included items related to predictability and reactivity, which Jones and Gosling [6] categorized into fearfulness. Most correlations between factors were small, as in previous studies [10,61], indicating good discriminant validity.

This study has limitations, especially regarding the broader use of the questionnaire. Firstly, dog owners reported their dog’s age, sex, and breed, and we did not confirm the accuracy of their answers. Secondly, the questionnaire has been translated to English, but the validity of this translated version has not been examined. Furthermore, all the respondents included in the test-retest and inter-rater reliability datasets were Finnish speakers residing in Finland. Therefore, before collecting behavioral data outside Finland, the reliability and validity of the translated questionnaire should be confirmed. The suitability of the questions about the living environment and background of the dog should be carefully assessed as well. Effort should also be made to ensure that dog owners understand their dog’s personality traits correctly. For example, dominance is a true concept, which is, however, often misunderstood by owners to mean that dogs would purposefully strive for dominance in dog-dog and dog-owner interactions [62].

In the future, we plan to analyze the dataset collected with this questionnaire, for example, examine the environmental factors associated with personality and unwanted behavior and the heritability of these traits. Behavior and personality traits are highly complex, as they are influenced by possibly hundreds of genes, tens of environmental factors in different life stages and interactions between these different factors [63], and behavior may change during life as well [64]. Therefore, longitudinal studies would be needed to assess the effect of these factors and trait change throughout life.

## 5. Conclusions

This study examined the structure, test-retest reliability, inter-rater reliability, convergent validity and discriminant validity of a dog personality and unwanted behavior questionnaire. We discovered that dog personality included seven personality traits, which paralleled previous dog personality traits. This personality and unwanted behavior questionnaire was shown to have good test-retest and inter-rater reliability. We examined the convergent validity with hypotheses formed based on previous research and most of these hypotheses held true, indicating excellent validity of this questionnaire tool. In conclusion, this questionnaire was shown to be a reliable and valid measure of dog personality and unwanted behavior.

## Figures and Tables

**Table 1:** Number of dogs in the whole datasets, and the number of dogs and time between answers in the test-retest reliability datasets and inter-rater reliability datasets.

	Whole Dataset	Test-Retest Reliability		Inter-Rater Reliability	
Section	*n*	*n*	Time between Answers	*n*	Time between Answers
Personality	12,865	129	Mean = 58 days (min 26 days, max 106 days)	73	Mean = 127 days (min 78 days, max 172 days)
Noise sensitivity	11,845	163	Mean = 58 days (min 22 days, max 106 days)	87	Mean = 122 days (min 78 days, max 188 days)
Fearfulness	11,995	146	Mean = 58 days (min 22 days, max 106 days)	79	Mean = 125 days (min 78 days, max 166 days)
Aggression	11,670	126	Mean = 59 days (min 26 days, max 106 days)	74	Mean = 126 days (min 78 days, max 166 days)
Fear of surfaces and heights	9946	108	Mean = 58 days (min 29 days, max 106 days)	54	Mean = 127 days (min 78 days, max 166 days)
Separation anxiety	10,511	118	Mean = 58 days (min 26 days, max 106 days)	62	Mean = 125 days (min 78 days, max 166 days)
Impulsivity/inattention	10,726	125	Mean = 58 days (min 26 days, max 106 days)	73	Mean = 125 days (min 78 days, max 166 days)

**Table 2:** Item loadings in the personality questionnaire.

Item	Insecurity	Training Focus	Energy	Aggressiveness/Dominance	Human Sociability	Dog Sociability	Perseverance
Erratic	**0.33**	**−0.45**	0.09	0.12	−0.09	−0.06	0.15
Aggressive to people	**0.34**	−0.02	0.02	**0.43**	−0.23	0.06	0.23
Sensitive to touch	**0.36**	−0.01	0.11	0.01	**−0.44**	−0.06	0.14
Human dependent	**0.37**	−0.05	−0.05	−0.07	0.28	−0.07	−0.15
Fearful of dogs	**0.56**	0	0.04	0.23	0.12	−0.24	−0.03
Cautious	**0.71**	0.18	−0.23	−0.15	0.05	0.02	0.01
Insecure	**0.89**	0.02	−0.02	−0.04	0.04	0.02	0.01
Anxious	**0.88**	−0.04	0	0.01	−0.02	−0.02	0.04
Fearful of people	**0.8**	0.1	0.01	0.08	−0.22	0.05	0.12
Wary	**0.71**	−0.05	−0.02	0.12	−0.01	0.01	0.13
Flexible	**−0.41**	**0.37**	−0.11	−0.1	0.11	0.04	0.07
Easily recovered	**−0.45**	**0.31**	−0.07	−0.02	0.06	0.09	0.09
Easygoing	**−0.66**	0.18	−0.1	−0.06	0.07	0.07	0.08
Bold	**−0.83**	0.06	0.04	0.05	−0.01	0	0.17
Confident	**−0.83**	0.03	−0.03	0.07	−0.02	0.04	0.19
Curious	**−0.38**	0.04	0.24	0	0.16	0.29	0.21
Independent	**−0.38**	0.2	−0.14	−0.1	−0.15	−0.1	0.28
Obedient	0.06	**0.67**	**0.31**	−0.02	0.11	−0.06	−0.16
Willing to learn	0.01	**0.46**	**0.51**	0	0.09	−0.04	0.05
Patient	−0.04	**0.57**	**−0.33**	−0.02	−0.01	0.11	−0.04
Calm	−0.13	**0.4**	**−0.51**	−0.24	−0.01	−0.02	0.06
Empathic	0.09	**0.3**	−0.08	0.03	**0.43**	0.05	0.09
Predictable	−0.07	**0.46**	−0.21	−0.04	0.09	0.02	−0.03
Reliable	−0.23	**0.48**	−0.07	−0.24	0.1	−0.1	0.01
Attentive	0.17	**0.49**	0.27	0.05	0.24	0.01	0.13
Focused	−0.11	**0.71**	0.02	−0.01	−0.06	−0.15	0.16
Intelligent	0.11	**0.46**	0.19	0.03	0.05	0.02	0.24
Restless	0.12	**−0.47**	**0.5**	−0.1	−0.02	−0.1	0.11
Excitable	0.17	**−0.41**	**0.52**	0.08	0.08	0.01	0.11
Provocative	0.01	**−0.32**	0.12	0.33	0	0.14	**0.35**
Stubborn	−0.1	**−0.48**	−0.09	0.04	−0.03	−0.01	**0.57**
Distractible	0.14	**−0.69**	0.04	−0.01	0.1	0.1	−0.05
Impulsive	0.13	**−0.52**	0.29	0.15	−0.03	0.02	0.15
Playful with people	−0.15	0.06	**0.33**	−0.01	**0.37**	0.19	0.05
Energetic	−0.06	0.08	**0.83**	−0.05	0.05	0.05	0.1
Boisterous	−0.04	−0.17	**0.42**	0.13	0.18	0.2	0.13
Active	−0.02	−0.07	**0.71**	−0.09	0.04	0.05	0.13
Playful alone	0.06	0.07	**0.32**	−0.03	0.15	0.27	0.15
Slow	0.04	0.08	**−0.72**	−0.03	0.06	0.01	0.08
Lazy	0.08	−0.22	**−0.77**	0.02	0.06	−0.14	0.14
Aggressive to dogs (same gender)	0.03	0.04	−0.05	**0.94**	0.03	0	−0.04
Aggressive to dogs (opposite gender)	0.1	0	−0.03	**0.78**	0.06	−0.12	−0.02
Dominant	−0.06	−0.02	−0.02	**0.8**	0.02	−0.01	0.11
Territorial	0.24	0.13	−0.01	**0.4**	−0.1	0.04	0.24
Sociable with dogs (same gender)	0.04	−0.02	−0.02	**−0.58**	0.07	**0.43**	0.12
Submissive	0.27	−0.04	0	**−0.72**	0.1	−0.02	−0.01
Calming	0.06	0.25	−0.16	**−0.33**	0.06	0.13	0.12
Human oriented	0.03	0.04	−0.04	0.1	**0.5**	**−0.63**	0.04
Sociable with people	−0.14	−0.04	0.01	−0.03	**0.84**	0.04	−0.03
Affectionate with people	0.11	0.01	−0.03	0	**0.73**	0.03	0.07
Attention seeking	0.17	−0.24	0.13	−0.03	**0.34**	−0.02	0.24
Solitary	0.1	−0.01	−0.23	0.01	**−0.39**	**−0.33**	0.11
Playful with dogs	−0.03	0.04	0.12	−0.15	0.07	**0.69**	0.04
Affectionate with dogs	0.07	0.02	−0.04	−0.1	0.23	**0.46**	0.06
Sociable with dogs (opposite gender)	−0.08	0.01	−0.06	−0.19	0.06	**0.65**	0.08
Indifferent	−0.03	0.16	−0.06	−0.11	−0.09	**−0.72**	0.15
Decisive	−0.19	0.25	−0.02	0.06	0.05	0.01	**0.58**
Persevering	−0.12	0.07	0.17	0.01	0.04	−0.05	**0.61**

Loadings >0.30 and <−0.30 are in bold.

**Table 3:** Internal consistency, test-retest reliability and inter-rater reliability of personality and unwanted behavior factors. ICC = Intraclass Correlation Coefficient.

		Internal Consistency		Test-Retest Reliability	Inter-Rater Reliability	
Section	Factor	Cronbach’s Alpha	Guttman’s Lambda 6	Correlation	ICC(1,1)	ICC(1,*k*)
Personality	Insecurity	0.89	0.91	0.91	0.72	0.84
	Training focus	0.87	0.90	0.91	0.70	0.82
	Energy	0.82	0.86	0.89	0.71	0.83
	Aggressiveness/dominance	0.80	0.84	0.91	0.81	0.90
	Human sociability	0.63	0.69	0.79	0.48	0.65
	Dog sociability	0.78	0.81	0.87	0.68	0.81
	Perseverance	0.61	0.67	0.70	0.52	0.68
Noise sensitivity	Noise sensitivity	0.95	0.97	0.93	0.80	0.89
Fearfulness	Fearfulness	0.90	0.92	0.89	0.74	0.85
Aggression	Barking	0.77	0.80	0.88	0.77	0.87
	Stranger directed aggression	0.74	0.79	0.83	0.69	0.82
	Owner directed aggression	0.63	0.68	0.82	0.72	0.84
	Dog directed aggression	0.69	0.69	0.87	0.66	0.80
Fear of surfaces	Fear of surfaces	0.77	0.77	0.80	0.74	0.85
Separation anxiety	Separation anxiety	0.76	0.79	0.78	0.50	0.66
Impulsivity/	Inattention	0.84	0.83	0.79	0.68	0.81
inattention	Hyperactivity/impulsivity	0.79	0.77	0.77	0.77	0.87
**Mean**		0.78	0.81	0.84	0.69	0.81

**Table 4:** Hypotheses formed to examine the convergent validity of the questionnaire, and their Pearson correlation coefficients, *t*-test statistics, sample sizes, and *p*-values. All *p*-values were corrected for false discovery rate (FDR).

Factor	Hypothesis	Test	Statistic	*n*	*p*-Value
Personality					
Energy	Older dogs less energetic	correlation	−0.33	12,865	<0.0001
	Belgian Shepherd Dogs, German Shepherd Dog and Australian Shepherd more active than Bernese Mountain Dogs, Mastiff-type dogs, brachycephalic dogs and teacup dogs	*t*-test	18.18, df = 1654.6	1837	<0.0001
Insecurity	Fearful dogs more insecure	correlation	0.75	10,622	<0.0001
	Large dogs less insecure	correlation	−0.13	11,406	<0.0001
Aggressiveness/dominance	Male dogs more aggressive	*t*-test	9.49, df = 12,697	12,865	<0.0001
	Dachshunds, German Shepherd Dog, teacup dogs and mixed breed dogs more aggressive/dominant than Bernese Mountain Dogs, Golden Retriever and Labrador Retriever	*t*-test	20.37, df = 2041.9	2044	<0.0001
Human sociability	Dogs with high training focus more sociable	correlation	0.12	12,865	<0.0001
	Insecure dogs less sociable	correlation	−0.13	12,865	<0.0001
	**Not met:** Large dogs more sociable	correlation	−0.09	11,406	<0.0001
Dog sociability	Older dogs less sociable	correlation	−0.47	12,865	<0.0001
	Aggressive/dominant dogs less sociable	correlation	−0.34	12,865	<0.0001
	Dogs high in dog-directed aggression less sociable	correlation	−0.33	10,538	<0.0001
Training focus	Older dogs more focused	correlation	0.13	12,865	<0.0001
	Australian Shepherd, Belgian Shepherd Dogs, German Shepherd Dog, Shetland Sheepdog and Poodles more focused than Dachshunds, sled dogs, brachycephalic dogs and teacup dogs	*t*-test	6.55, df = 1301.1	2310	<0.0001
Perseverance	Insecure dogs less persevering	correlation	−0.13	12,865	<0.0001
	Energetic dogs more persevering	correlation	0.15	12,865	<0.0001
Fearfulness	Female dogs more fearful	*t*-test	8.40, df = 11,986	11,995	<0.0001
	Jack Russell Terrier, Lagotto Romagnolo, Shetland Sheepdog, teacup dogs and mixed breed dogs more fearful than Bull-type terriers, Golden Retriever, Labrador Retriever and German Shepherd Dog	*t*-test	17.40, df = 2233.2	2437	<0.0001
	Dogs classified as having fear of strangers, dogs or situations more fearful than dogs classified as non-fearful	*t*-test	75.31, df = 5730.5	11,219	<0.0001
Noise sensitivity	Older dogs more fearful of noises	correlation	0.18	11,844	<0.0001
	Fears of different noises correlate *	-	−	-	-
	Large dogs less fearful of noises	correlation	−0.09	11,329	<0.0001
	Dogs classified as having fear of thunder, firework or other noises more fearful than dogs classified as non-fearful	*t*-test	65.44, df = 4499.2	11,842	<0.0001
Separation anxiety	Dogs fearful of noises have more separation anxiety	correlation	0.19	9446	<0.0001
	**Not met:** Large dogs have less separation anxiety	correlation	−0.006	10,127	0.573
	Dogs classified as separation anxious have more separation anxiety	*t*-test	31.48, df = 1434.3	10,296	<0.0001
**Aggression**					
Barking	**Not met:** Male dogs bark more	*t*-test	−1.43, df = 11,515	11,670	0.157
	Dogs aggressive towards strangers bark more	correlation	0.61	11,670	<0.0001
	Dogs classified as aggressive towards strangers bark more	*t*-test	36.47, df = 671.09	11,384	<0.0001
Stranger directed aggression	Older dogs more aggressive	correlation	0.07	11,670	<0.0001
	Male dogs more aggressive	*t*-test	3.88, df = 11,194	11,670	0.0001
	Fearful dogs more aggressive	correlation	0.39	11,040	<0.0001
	Dogs classified as aggressive towards strangers more aggressive	*t*-test	32.38, df = 649.02	11,384	<0.0001
Dog directed aggression	Older dogs more aggressive	correlation	0.20	11,668	<0.0001
	Fearful dogs more aggressive	correlation	0.26	11,040	<0.0001
	Dachshunds, German Shepherd Dogs and teacup dogs more aggressive than Bernese Mountain Dogs, Golden Retriever and Labrador Retriever	*t*-test	13.42, df = 1270.7	1462	<0.0001
	Dogs classified as aggressive towards dogs more aggressive	*t*-test	66.54, df = 3569.6	11,161	<0.0001
Owner directed aggression	**Not met:** older dogs more aggressive	correlation	0.01	11,670	0.143
	Male dogs more aggressive	*t*-test	5.53, df = 11,085	11,670	<0.0001
	Dogs aggressive towards strangers more aggressive towards the owner	correlation	0.34	11,670	<0.0001
	Dogs classified as aggressive towards the owner more aggressive	*t*-test	18.52, df = 277.11	11,519	<0.0001
Fear of surfaces and heights	Fearful dogs more fearful of surfaces	correlation	0.22	9601	<0.0001
	Dogs classified as fearful of surfaces more fearful	*t*-test	40.38, df = 963.54	9754	<0.0001
**Impulsivity/inattention**					
Hyperactivity/impulsivity	Older dogs less impulsive	correlation	−0.15	10,726	<0.0001
	Dogs with high training focus less impulsive	correlation	−0.63	10,194	<0.0001
	Dogs classified as impulsive more hyperactive/impulsive	*t*-test	58.10, df = 2494.4	10,168	<0.0001
	Dogs of owners more disturbed by impulsive behavior more hyperactive/impulsive	correlation	0.69	10,657	<0.0001
Inattention	Older dogs less inattentive	correlation	−0.10	10,726	<0.0001
	Dogs with high training focus less inattentive	correlation	−0.69	10,194	<0.0001
	Dogs classified as impulsive more inattentive	*t*-test	33.33, df = 2656	10,168	<0.0001
	Dogs of owners more disturbed by impulsive behavior more inattentive	correlation	0.49	10,657	<0.0001

* Behavioral reactions toward different noises loaded onto the same factor, indicating a high correlation between fear of different noises. As a result, statistical analysis was not possible.

**Table 5:** Factor correlations in the personality questionnaire.

	Insecurity	Training Focus	Energy	Aggressiveness/Dominance	Human Sociability	Dog Sociability	Perseverance
Insecurity	1	**−0.30**	0.07	0.18	−0.13	−0.18	−0.13
Training focus	**−0.30**	1	−0.17	−0.23	0.12	−0.06	−0.04
Energy	0.07	−0.17	1	0.13	0.17	0.22	0.15
Aggressiveness/dominance	0.18	−0.23	0.13	1	−0.13	**−0.34**	0.17
Human sociability	−0.13	0.12	0.17	−0.13	1	0.11	0.00
Dog sociability	−0.18	−0.06	0.22	**−0.34**	0.11	1	0.07
Perseverance	−0.13	−0.04	0.15	0.17	0.00	0.07	1

Moderate correlations (>0.3) are in bold.

## Data Availability

The data presented in this study are openly available in FigShare: https://figshare.com/articles/dataset/Salonen_et_al_Reliability_and_Validity_of_a_Dog_Personality_and_Unwanted_Behavior_Survey/14479152 (accessed on 24 April 2021).

## Supplementary Materials

The following are available online at https://www.mdpi.com/article/10.3390/ani11051234/s1, Table S1. Breeds and breed groups, Table S2. Hypotheses and studies they were derived from, Table S3. Item loadings, test-retest reliabilities and inter-rater reliabilities in the noise sensitivity questionnaire, Table S4. Item loadings, test-retest reliabilities and inter-rater reliabilities in the fearfulness questionnaire, Table S5. Item loadings, test-retest reliabilities and inter-rater reliabilities in the fear of surfaces and heights questionnaire, Table S6. Item loadings, test-retest reliabilities and inter-rater reliabilities in the separation anxiety questionnaire, Table S7. Item loadings, test-retest reliabilities and inter-rater reliabilities in the impulsivity/inattention questionnaire. Questionnaire translated from [50], Table S8. Item loadings, test-retest reliabilities and inter-rater reliabilities in the aggression questionnaire, Table S9. Test-retest reliabilities and inter-rater reliabilities in the personality questionnaire, Supplementary File: Questionnaire.
