# Supplementary material for: Reliability and Validity of a Dog Personality and Unwanted Behavior Survey
Source: Animals (Basel). 2021 Apr 24;11(5):1234. doi: 10.3390/ani11051234 (PMC8147106; doi:10.3390/ani11051234)
Supplement: Supplementary file 1 [file animals-11-01234-s001.zip › animals-1179236-SI.pdf]

animals

MDPI

Article

Reliability and Validity of a Dog Personality and Unwanted Behavior Survey

Milla Salonen [1,2,3], Salla Mikkola [1,2,3], Emma Hakanen [1,2,3], Sini Sulkama [1,2,3], Jenni Puurunen [1,2,3] and Hannes Lohi [1,2,3,*]

[1] Department of Veterinary Biosciences, University of Helsinki, 00014 Helsinki, Finland; milla.ahola@helsinki.fi (M.S.); salla.mikkola@helsinki.fi (S.M.); emma.hakanen@helsinki.fi (E.H.); sini.sulkama@helsinki.fi (S.S.); jenni.puurunen@petbiomics.com (J.P.)
[2] Department of Medical and Clinical Genetics, University of Helsinki, 00014 Helsinki, Finland
[3] Folkhälsan Research Center, 00290 Helsinki, Finland
* Correspondence: hannes.lohi@helsinki.fi

Simple Summary: Dogs have distinct personality, meaning differences between individuals that persist throughout their lives. However, it is still unclear what traits are required to define the whole personality of dogs. Personality and unwanted behavior are often studied using behavioral questionnaires, but researchers should ensure that these questionnaires are reliable and valid, meaning that they measure the behavior traits they were intended to measure. In this study, we first examined what traits define a dog's personality. We discovered seven personality traits: Insecurity, Training focus, Energy, Aggressiveness/dominance, Human sociability, Dog sociability, and Perseverance. We also studied six unwanted behavior traits: noise sensitivity, fearfulness, aggression (including barking, stranger directed aggression, owner directed aggression and dog directed aggression), fear of surfaces and heights, separation anxiety, and impulsivity/inattention (including hyperactivity/impulsivity and inattention). We examined the reliability of these traits by asking some dog owners to answer to the questionnaire twice, several weeks apart, and by asking another family member to answer the questionnaire of the same dog. Furthermore, we studied the validity of these traits by forming predictions based on previous literature. Based on our results, this personality and unwanted behavior questionnaire is a good tool to study dog behavior.

Citation: Salonen, M.; Mikkola, S.; Hakanen, E.; Sulkama, S.; Puurunen, J.; Lohi, H. Reliability and validity of a dog personality and unwanted behavior survey. *Animals* **2021**, *11*, 1234. https://doi.org/10.3390/ani11051234

Academic Editors Sylvia García-Belenguer

Received: 26 March 2021
Accepted: 21 April 2021
Published: 24 April 2021

Publisher's Note: MDPI stays neutral with regard to jurisdictional claims in published maps and institutional affiliations.

Copyright: © 2021 by the authors. Licensee MDPI, Basel, Switzerland. This article is an open access article distributed under the terms and conditions of the Creative Commons Attribution (CC BY) license (http://creativecommons.org/licenses/by/4.0/).

Table S1. Breeds and breed groups.

**Australian Shepherd**<br>Australian Shepherd<br>Miniature American Shepherd	**Belgian Shepherd Dog**<br>Groenendael<br>Laekenois<br>Malinois<br>Tervueren	**Bernese Mountain Dogs**<br>Appenzell Cattle Dog<br>Bernese Mountain Dog<br>Entlebucher Mountain Dog<br>Greater Swiss Mountain Dog	**Bichon Type Dogs**<br>Bichon Frise<br>Bolognese<br>Coton de Tulear<br>Havanese<br>Löwchen<br>Maltese<br>Russian Tsvetnaya Bolonka
**Brachycephalic Dogs**<br>Boston Terrier<br>Bulldog<br>French Bulldog<br>Olde English Bulldogge<br>Pug	**Bull Type Terriers**<br>Bull Terrier<br>Miniature Bull Terrier<br>Staffordshire Bull Terrier	**Dachshunds**<br>Miniature Longhaired<br>Miniature Shorthaired<br>Miniature Wirehaired<br>Rabbit Longhaired<br>Rabbit Shorthaired<br>Rabbit Wirehaired<br>Standard Longhaired<br>Standard Shorthaired<br>Standard Wirehaired	**English Herders**<br>Australian Cattle Dog<br>Australian Kelpie<br>Australian Koolie<br>Bearded Collie<br>Lancashire Heeler<br>Old English Sheepdog<br>Welsh Sheepdog
**European Sighthounds**<br>Borzoi<br>Deerhound<br>Greyhound<br>Hungarian Greyhound<br>Irish Wolfhound<br>Italian Sighthound<br>Polish Greyhound<br>Silken Windhound<br>Spanish Greyhound	**Fighting Dogs**<br>American Bulldog<br>American Bully<br>American Pit Bull Terrier<br>American Staffordshire Terrier<br>Cane Corso<br>Dogo Argentino<br>Presa Canario	**German Spitz Related**<br>Eurasier<br>Giant Spitz<br>Medium size Spitz<br>Miniature Spitz<br>Japanese Spitz<br>Keeshond<br>Pomeranian<br>Volpino Italiano	**Hunting Terriers**<br>Bedlington Terrier<br>Border Terrier<br>Cairn Terrier<br>Cesky Terrier<br>Dandie Dinmont Terrie<br>Fox Terrier Smooth<br>Fox Terrier Wire<br>German Hunting Terrie<br>Glen of Imaal Terrier<br>Lakeland Terrier<br>Norfolk Terrier<br>Norwich Terrier<br>Scottish Terrier<br>Skye Terrier<br>Welsh Terrier<br>West Highland White Terrier
**Asian Primitive Dogs**<br>Akita<br>American Akita<br>Chow Chow<br>Hokkaido<br>Kai<br>Kishu<br>Korea Jindo Dog<br>Shar Pei<br>Shiba<br>Shikoku<br>Tosa	**Livestock Guardian Dogs**<br>Boerboel<br>Catalan Sheepdog<br>Caucasian Shepherd Dog<br>Central Asian Shepherd Dog<br>Estrela Mountain Dog<br>Maremma Sheepdog<br>Pyrenean Mastiff<br>Pyrenean Mountain Dog<br>Sarplaninac<br>Slovakian Chuvach<br>South Russian Ovcharka<br>Spanish Mastiff<br>Tatra Shepherd Dog<br>Tibetan Mastiff<br>Tornjak	**Mastiff Type Dogs**<br>Boxer<br>Broholmer<br>Bullmastiff<br>Dogue de Bordeaux<br>Great Dane<br>Landseer<br>Leonberger<br>Mastiff<br>Newfoundland<br>Saint Bernard Longhaired<br>Saint Bernard Shorthaired	**Middle European Herders**<br>Beauceron<br>Bouvier des Ardennes<br>Bouvier des Flandres<br>Briard<br>Croatian Sheepdog<br>Mudi<br>Picardy Sheepdog<br>Polish Lowland Sheepdog<br>Portuguese Sheepdog<br>Puli<br>Pumi<br>Pyrenean Sheepdog Longhaired<br>Pyrenean Sheepdog Smooth Faced
**Middle European Utility Dogs**<br>Bohemian Shepherd	**Northern Companion Spitz**<br>Icelandic Sheepdog<br>Norwegian Buhund	**Northern Hunting Spitz**<br>East Siberian Laika<br>Finnish Spitz	**Other Breed**<br>Australian Cobberdog<br>Australian Labradoodle

Ceskoslovensky Vlcak<br>Dalmatian<br>Dobermann<br>Dutch Shepherd Dog Longhaired<br>Dutch Shepherd Dog Rough haired<br>Dutch Shepherd Dog Shorthaired<br>East European Shepherd<br>Hovawart<br>Rottweiler<br>Saarloos Wolfdog<br>Schipperke	Swedish Lapphund<br>Swedish Vallhund	Karelian Bear Dog<br>Norrbottenspitz<br>Norwegian Elkhound<br>Russian European Laika<br>Swedish Elkhound<br>West Siberian Laika	Golden Labrador Retriever<br>Indian Pariah Dog<br>Peruvian Hairless Dog Large<br>Peruvian Hairless Dog Medium sized<br>Peruvian Hairless Dog Miniature<br>Seskar seal dog<br>Unknown breed<br>Wolfdog<br>Xoloitzcuintle Intermediate<br>Xoloitzcuintle Standard
**Other Companion Dogs**<br>Affenpinscher<br>Alaskan Klee Kai<br>Australian Silky Terrier<br>Cavalier King Charles Spaniel<br>Griffon Belge<br>Griffon Bruxellois<br>King Charles Spaniel<br>Kromfohrlander<br>Lhasa Apso<br>Papillon<br>Pekingese<br>Petit Brabancon<br>Phalene<br>Shih Tzu<br>Tibetan Spaniel<br>Tibetan Terrier	**Parson Type Terriers**<br>Brazilian terrier<br>Parson Russell Terrier<br>Working Jack Russell Terrier	**Pinschers and Schnauzers**<br>Black Russian Terrier<br>German Pinscher<br>Giant Schnauzer<br>Schnauzer	**Pointers**<br>Blue Picardy Spaniel<br>Braque du Bourbonnais<br>Brittany<br>Deutsch Drahthaar<br>Deutsch Kurzhaar<br>Deutsch Langhaar<br>English Pointer<br>English Setter<br>Gordon Setter<br>Irish Red and White Setter<br>Irish Red Setter<br>Large Munsterlander<br>Old Danish Pointer<br>Pont Audemer Spaniel<br>Portuguese Pointer<br>Small Munsterlander<br>Spinone Italiano<br>Stabyhoun<br>Vizsla<br>Weimaraner Longhaired<br>Weimaraner Shorthaired<br>Wirehaired Pointing Griffon<br>Wirehaired Vizsla
**Poodles**<br>Poodle Medium<br>Poodle Miniature<br>Poodle Standard<br>Poodle Toy	**Primitive Sighthounds**<br>Afghan Hound<br>Andalusian Hound<br>Basenji<br>Cirneco dell'Etna<br>Ibizan Hound Smooth<br>Ibizan Hound Wire<br>Pharaoh Hound<br>Podenco Orito<br>Portuguese Podengo Medium<br>Portuguese Podengo Medium Smooth<br>Portuguese Podengo Medium Wire	**Retrievers/Flushing Dogs**<br>American Cocker Spaniel<br>American Water Spaniel<br>Barbet<br>Chesapeake Bay Retriever<br>Clumber Spaniel<br>Curly Coated Retriever<br>English Cocker Spaniel<br>English Springer Spaniel<br>Field Spaniel<br>Flat Coated Retriever<br>Irish Water Spaniel<br>Kooikerhondje	**Scenthounds**<br>American Foxhound<br>Barak Hound<br>Basset Fauve de Bretagne<br>Basset Hound<br>Bavarian Mountain Hound<br>Beagle<br>Bracco Italiano<br>Braque d'Auvergne<br>Drever<br>Estonian Hound<br>Finnish Hound<br>Grand Basset Griffon Vendeen
	Portuguese Podengo Miniature Smooth<br>Portuguese Podengo Miniature Wire	Portuguese Water Dog<br>Welsh Springer Spaniel	Griffon Fauve de Bretagne<br>Istrian Coarse-haired Hound<br>Italian Rough Haired Segugio<br>Italian Short Haired Segugio<br>Petit Basset Griffon Vendeen

	Ratonero Bodeguero Andaluz<br>Rhodesian Ridgeback<br>Saluki<br>Sloughi<br>Thai Ridgeback		Plott<br>Posavatz Hound<br>Russian Hound<br>Swiss Hound
**Sled Dogs**<br>Alaskan Husky<br>Alaskan Malamute<br>Chukotka Sled Dog<br>Greenland Dog<br>Samoyed<br>Siberian Husky<br>Yakutian Laika	**Teacup Dogs**<br>Biewer Terrier<br>Chihuahua<br>Chihuahua Longhaired<br>Chihuahua Smooth haired<br>Russian Toy Dog Longhaired<br>Russian Toy Dog Smooth haired<br>Yorkshire Terrier	**Welsh Corgis**<br>Welsh Corgi Cardigan<br>Welsh Corgi Pembroke	**Yard Terriers**<br>Airedale Terrier<br>American Hairless Terrier<br>Australian Terrier<br>Danish Swedish Farmdog<br>English Toy Terrier<br>Irish Soft Coated Wheaten Terrier<br>Irish Terrier<br>Kerry Blue Terrier<br>Manchester Terrier<br>Prague Ratter<br>Rat Terrier<br>Tenterfield Terrier<br>Toy Fox Terrier
**Individual Breeds**<br>Border Collie<br>Chinese Crested Dog<br>Collie Rough<br>Collie Smooth<br>Finnish Lapphund<br>German Shepherd Dog	Golden Retriever<br>Jack Russell Terrier<br>Labrador Retriever<br>Lagotto Romagnolo<br>Lapponian Herder<br>Miniature Pinscher	Miniature Schnauzer<br>Mixed breed<br>Nova Scotia Duck Tolling Retriever<br>Schapendoes<br>Shetland Sheepdog	Spanish Water Dog<br>Whippet<br>White Swiss Shepherd Dog

Table S2. Hypotheses and studies they were derived from.

Factor	Hypothesis	Reference
**Personality**		
Energy	Older dogs less energetic	[1–4]
	Belgian Shepherd Dogs, German Shepherd Dog and Australian Shepherd more active than Bernese Mountain Dogs, Mastiff-type dogs, brachycephalic dogs and teacup dogs	[5,6]
Insecurity	Fearful dogs more insecure	[7]
	Large dogs less insecure	[2,3,8]
Aggressiveness/dominance	Male dogs more aggressive	[8–11]
	Dachshunds, German Shepherd Dog, teacup dogs and mixed breed dogs more aggressive/dominant than Bernese Mountain Dogs, Golden Retriever and Labrador Retriever	[5,12,13]
Human sociability	Dogs with high training focus more sociable	[2,3]
	Insecure dogs less sociable	[2,3]
	Large dogs more sociable	[2]
Dog sociability	Older dogs less sociable	[14]
	Aggressive/dominant dogs less sociable	[5]
	Dogs high in dog-directed aggression less sociable	[5]
Training focus	Older dogs more focused	[2,4,14]
	Australian Shepherd, Belgian Shepherd Dogs, German Shepherd Dog, Shetland Sheepdog and Poodles more focused than Dachshunds, sled dogs, brachycephalic dogs and teacup dogs	[5,6]
Perseverance	Insecure dogs less persevering	[2]
	Energetic dogs more persevering	[2]
**Fearfulness**	Female dogs more fearful	[3,5,9,14–16]
	Jack Russell Terrier, Lagotto Romagnolo, Shetland Sheepdog, teacup dogs and mixed breed dogs more fearful than Bull-type terriers, Golden Retriever, Labrador Retriever and German Shepherd Dog	[3,5,6,12,13,15,16]
	Dogs classified as having fear of strangers, dogs or situations more fearful than dogs classified as non-fearful	-
**Noise sensitivity**	Older dogs more fearful of noises	[17–19]
	Fears of different noises correlate	[11,18,19]
	Large dogs less fearful of noises	[8,16]
	Dogs classified as having fear of thunder, firework or other noises more fearful than dogs classified as non-fearful	-
**Separation anxiety**	Dogs fearful of noises have more separation anxiety	[13,17]
	Large dogs have less separation anxiety	[8,16,20]
	Dogs classified as separation anxious have more separation anxiety	-
**Aggression**		
Barking	Male dogs bark more	[9]
	Dogs aggressive towards strangers bark more	[11]
	Dogs classified as aggressive towards strangers bark more	-
Stranger directed aggression	Older dogs more aggressive	[21,22]
	Male dogs more aggressive	[3,11,23]
	Fearful dogs more aggressive	[3,5,13,23]
	Dogs classified as aggressive towards strangers more aggressive	-
Dog directed aggression	Older dogs more aggressive	[21,22]
	Fearful dogs more aggressive	[5,13]
	Dachshunds, German Shepherd Dogs and teacup dogs more aggressive than Bernese Mountain Dogs, Golden Retriever and Labrador Retriever	[5,12]
	Dogs classified as aggressive towards dogs more aggressive	-
Owner directed aggression	Older dogs more aggressive	[10,21]
	Male dogs more aggressive	[3,10,11]
	Dogs aggressive towards strangers more aggressive towards the owner	[10]

	Dogs classified as aggressive towards the owner more aggressive	-
**Fear of surfaces and heights**	Fearful dogs more fearful of surfaces	[24]
	Dogs classified as fearful of surfaces more fearful	-
**Impulsivity/ inattention**		
Hyperactivity/ impulsivity	Older dogs less impulsive	[2,14,25–27]
	Dogs with high training focus less impulsive	-
	Dogs classified as impulsive more hyperactive/impulsive	-
	Dogs of owners more disturbed by impulsive behavior more hyperactive/ impulsive	-
Inattention	Older dogs less inattentive	[2,14,25–27]
	Dogs with high training focus less inattentive	-
	Dogs classified as impulsive more inattentive	-
	Dogs of owners more disturbed by impulsive behavior more inattentive	-

Table S3. Item loadings, test-retest reliabilities and inter-rater reliabilities in the noise sensitivity questionnaire.

Items	Loadings	Test-Retest Reliability	Interrater Reliability	
	Noise Sensitivity	Correlation	ICC(1,1)	ICC(1,*k*)
Thunder: escapes	**0.87**	0.85	0.58	0.73
Thunder: pants	**0.86**	0.85	0.68	0.81
Thunder: hides	**0.85**	0.87	0.71	0.83
Thunder: trembles	**0.9**	0.81	0.72	0.83
Thunder: paces	**0.77**	0.69	0.55	0.71
Thunder: tail low or between legs	**0.86**	0.8	0.68	0.81
Thunder: freezes	**0.69**	0.7	0.21	0.35
Thunder: vocalizes	**0.6**	0.54	0.48	0.65
Thunder: salivates	**0.74**	0.77	0.76	0.86
Thunder: stays close to the owner	**0.62**	0.73	0.28	0.44
Thunder: indifferent	**− 0.81**	0.72	0.61	0.76
Thunder: notices the sound but continues what was doing	**− 0.69**	0.54	0.60	0.75
Fireworks: escapes	**0.84**	0.81	0.55	0.71
Fireworks: pants	**0.87**	0.82	0.71	0.83
Fireworks: hides	**0.84**	0.81	0.84	0.91
Fireworks: trembles	**0.89**	0.89	0.71	0.83
Fireworks: paces	**0.76**	0.63	0.59	0.74
Fireworks. tail low or between legs	**0.88**	0.87	0.70	0.82
Fireworks: freezes	**0.67**	0.75	0.43	0.61
Fireworks: vocalizes	**0.59**	0.68	0.40	0.57
Fireworks: salivates	**0.76**	0.65	0.65	0.78
Fireworks: stays close to the owner	**0.62**	0.72	0.27	0.43
Fireworks: indifferent	**−0.82**	0.81	0.65	0.79
Fireworks: notices the sound but continues what was doing	**−0.79**	0.74	0.71	0.83
Other sounds: escapes	**0.5**	0.62	0.23	0.38
Other sounds: pants	**0.66**	0.65	0.49	0.66
Other sounds: hides	**0.59**	0.74	0.61	0.76
Other sounds: trembles	**0.71**	0.64	0.71	0.83
Other sounds: paces	**0.51**	0.63	0.48	0.65
Other sounds: tail low or between legs	**0.66**	0.71	0.55	0.71
Other sounds: freezes	**0.48**	0.68	0.34	0.51
Other sounds: vocalizes	**0.35**	0.59	0.29	0.45
Other sounds: stays close to the owner	**0.47**	0.61	0.44	0.61
Other sounds: indifferent	**−0.53**	0.53	0.40	0.58
Other sounds: notices the sound but continues what was doing	**−0.5**	0.47	0.32	0.48
REMOVED ITEMS				
Thunder: barks or growls [1]	-	0.65	0.55	0.71
Firework: barks or growls [1]	-	0.8	0.50	0.66
Other sounds: barks or growls [1]	-	0.63	0.57	0.73
Other sounds: salivates [2]	-	0.34	0.14	0.25

[1]Did not load onto any factor. [2]low reliability. ICC = Intraclass Correlation Coefficient. Loadings > 0.30 and < -0.30 are in bold.

Table S4. Item loadings, test-retest reliabilities and inter-rater reliabilities in the fearfulness questionnaire.

Items	Loadings	Test-Retest Reliability	Inter-Rater Reliability	
	Fearfulness	Correlation	ICC(1,1)	ICC(1,*k*)
Strangers: withdraws	**0.8**	0.75	0.52	0.68
Strangers: barks	**0.45**	0.75	0.68	0.81
Strangers: growls	**0.54**	0.61	0.60	0.75
Strangers: tail low or between legs	**0.77**	0.67	0.55	0.71
Strangers: stays close to the owner	**0.56**	0.5	0.47	0.64
Strangers: not willing to make contact	**0.59**	0.42	0.54	0.70
Strangers: greets enthusiastically	**−0.55**	0.8	0.70	0.82
Strangers: approaches with a low posture	**0.65**	0.64	0.29	0.46
Strangers: moves away if the stranger tries to touch	**0.78**	0.76	0.53	0.69
Strangers: suspicious at the beginning	**0.77**	0.87	0.55	0.71
Unfamiliar dogs: growls	**0.3**	0.74	0.46	0.63
Unfamiliar dogs: tail low or between legs	**0.57**	0.57	0.56	0.72
Unfamiliar dogs: not willing to make contact	**0.39**	0.45	0.54	0.70
Unfamiliar dogs: enthusiastic	**−0.37**	0.71	0.67	0.80
Unfamiliar dogs: approaches with a low posture	**0.41**	0.6	0.41	0.58
Unfamiliar dogs: wants to escape	**0.53**	0.66	0.19	0.32
Unfamiliar dogs: suspicious at the beginning	**0.54**	0.58	0.33	0.50
New situations: tail low or between legs	**0.69**	0.6	0.47	0.64
New situations: pants	**0.41**	0.56	0.30	0.46
New situations: wants out of the situation/place	**0.66**	0.63	0.33	0.49
New situations: trembles	**0.55**	0.63	0.27	0.42
New situations: stays close to the owner	**0.54**	0.7	0.43	0.60
New situations: walks low to the ground	**0.63**	0.61	0.42	0.59
New situations: restless and can't calm down	**0.43**	0.65	0.51	0.67
New situations: curious and enthusiastic	**−0.6**	0.67	0.51	0.68
New situations: able to eat and sleep	**−0.45**	0.47	0.55	0.71
New situations: cautious at the beginning	**0.68**	0.71	0.36	0.53
REMOVED ITEMS				
Strangers: indifferent but doesn't try to move away [1]	-	0.53	0.37	0.54
Unfamiliar dogs: barks [1]	-	0.8	0.76	0.86
Unfamiliar dogs: indifferent but doesn't try to move away[1]	-	0.61	0.48	0.65
Unfamiliar dogs: tries to attack [1]	-	0.72	0.64	0.78

[1]Did not load onto any factor. ICC = Intraclass Correlation Coefficient. Loadings > 0.30 and < -0.30 are in bold.

Table S5. Item loadings, test-retest reliabilities and inter-rater reliabilities in the fear of surfaces and heights questionnaire.

Items	Loadings	Test-Retest Reliability	Inter-Rater Reliability	
**Difficulties**	**Fear of Surfaces**	**Correlation**	**ICC(1,1)**	**ICC(1,*k*)**
on a metal grid	**0.58**	0.7	0.39	0.56
on shiny floors	**0.94**	0.73	0.72	0.84
on open riser stairs	**0.68**	0.64	0.45	0.63
on closed riser_stairs	**0.72**	0.64	0.45	0.62
moving from one surface to another	**0.75**	0.64	0.34	0.51
on a slippery floor	**0.92**	0.64	0.56	0.72
on narrow bridges [1]	-	0.53	−0.07	−0.16

[1]low reliability. ICC = Intraclass Correlation Coefficient. Loadings > 0.30 and < -0.30 are in bold.

Table S6. Item loadings, test-retest reliabilities and inter-rater reliabilities in the separation anxiety questionnaire.

Items	Loadings	Test-Retest Reliability	Inter-Rater Reliability	
	**Separation Anxiety**	**Correlation**	**ICC(1,1)**	**ICC(1,*k*)**
Pants when owner is leaving	**0.92**	0.57	0.51	0.67
Vocalizes when owner is leaving	**0.69**	0.7	0.33	0.50
Salivates when owner is leaving	**0.86**	0.29	0.33	0.50
Restless when owner is leaving	**0.78**	0.67	0.52	0.69
Destroys when home alone	**0.37**	0.85	0.55	0.71
Urinates/defecates when home alone	**0.36**	0.74	0.70	0.82
Vocalizes when home alone	**0.6**	0.73	0.59	0.75
Pants when home alone	**0.86**	0.65	0.15	0.25
REMOVED ITEMS				
Salivates when home alone [1]	-	0.46	−0.01	−0.03

[1]low reliability. ICC = Intraclass Correlation Coefficient. Loadings > 0.30 and < -0.30 are in bold.

Table S7. Item loadings, test-retest reliabilities and inter-rater reliabilities in the impulsivity/inattention questionnaire. Questionnaire translated from [28].

Items	Loadings		Test-Retest Reliability	Inter-Rater Reliability	
	IA	H/I	Correlation	ICC(1,1)	ICC(1,*k*)
1. Difficulties learning as is careless/other things attract attention	**0.76**	0.13	0.71	0.55	0.71
2. Easy to attract attention but loses interest soon	**0.78**	−0.19	0.6	0.51	0.68
3. Difficult to concentrate on a task/play	**0.87**	−0.04	0.59	0.38	0.55
4. Leaves from place when should stay	**0.37**	0.26	0.63	0.28	0.44
5. Cannot be quiet or easily calmed	−0.06	**0.78**	0.64	0.76	0.86
6. Fidgets all the time	−0.01	**0.76**	0.47	0.52	0.69
7. Seems that doesn't listen	**0.46**	0.19	0.64	0.52	0.68
8. Is excessive and difficult to control	0.08	**0.74**	0.63	0.57	0.72
9. Would always play and run	−0.11	**0.58**	0.64	0.36	0.53
10. Difficulties with complicated tasks	**0.55**	0.11	0.42	0.36	0.53
11. Likely to react hastily	**0.33**	**0.47**	0.52	0.37	0.54
12. Attention can be easily distracted	**0.65**	0.21	0.72	0.31	0.47
13. Cannot wait as has no self-control	0.26	**0.57**	0.48	0.44	0.61

IA: Inattention; H/I: Hyperactivity/impulsivity. ICC = Intraclass Correlation Coefficient. Loadings > 0.30 and < -0.30 are in bold.

Table S8. Item loadings, test-retest reliabilities and inter-rater reliabilities in the aggression questionnaire.

Items	Loadings				Test-Retest Reliability	Inter-Rater Reliability	
	BARK	STR	OWN	DOG	Correlation	ICC(1,1)	ICC(1,*k*)
Barks at doorbell/knocking	**0.64**	−0.2	0.07	0.15	0.88	0.68	0.81
Barks when strangers come in	**0.87**	0.03	0.01	0.03	0.81	0.78	0.87
At home, barks when strangers try to touch	**0.93**	0.05	0.02	−0.06	0.7	0.55	0.71
At home, growls when strangers try to touch	**0.4**	**0.49**	0.03	0.16	0.63	0.62	0.76
At home, tries to snap/bite when strangers try to touch	0.07	**0.88**	0.09	0.01	0.75	0.56	0.72
Happy and excited when strangers come in	**−0.43**	**−0.33**	0.15	0.09	0.79	0.59	0.74
When leashed, barks when strangers try to touch	**0.74**	0.12	−0.01	0.04	0.72	0.59	0.75
When leashed, growls when strangers try to touch	0.28	**0.56**	−0.03	0.24	0.45	0.65	0.78
When leashed, tries to snap/bite when strangers try to touch	0.02	**0.91**	0.05	0.07	0.77	0.60	0.75
Barks/growls in the car at passersby	**0.36**	−0.01	0.06	0.22	0.64	0.43	0.60
Growls when handled by the owner	0.02	0.09	**0.48**	0.1	0.86	0.83	0.91
Tries to snap/bite when handled by the owner	−0.08	0.27	**0.59**	−0.13	0.86	0.63	0.77
Growls when owner tries to take away food/bone/toy	0.09	−0.15	**0.87**	0.1	0.78	0.67	0.80
Tries to snap/bite when owner tries to take away food/bone/toy	-0.03	0.1	**0.96**	−0.06	0.71	0.51	0.68
Growls when meets unfamiliar dogs	0.02	0.01	−0.03	**0.88**	0.74	0.58	0.73
Tries to attack when meets unfamiliar dogs	−0.1	0.25	0.01	**0.67**	0.8	0.77	0.87
Growls when familiar dogs approach food/toy/treat	0.09	−0.12	0.26	**0.43**	0.81	0.59	0.74
Tries to attack when familiar dogs approach food/toy/treat	0.01	0.05	**0.32**	**0.4**	0.77	0.45	0.62

BARK: Barking; STR: Stranger directed aggression; OWN: Owner directed aggression; DOG: Dog directed aggression. ICC = Intraclass Correlation Coefficient. Loadings > 0.30 and < -0.30 are in bold.

Table S9. Test-retest reliabilities and inter-rater reliabilities in the personality questionnaire.

Items	Test-Retest Reliability	Inter-Rater Reliability	
	Correlation	ICC(1,1)	ICC(1,*k*)
Erratic	0.56	0.34	0.51
Aggressive to people	0.87	0.75	0.86
Sensitive to touch	0.6	0.25	0.40
Human dependent	0.56	0.41	0.58
Fearful of dogs	0.84	0.48	0.65
Cautious	0.66	0.39	0.56
Insecure	0.75	0.45	0.62
Anxious	0.78	0.38	0.55
Fearful of people	0.82	0.79	0.88
Wary	0.65	0.36	0.53
Flexible	0.6	0.55	0.71
Easily recovered	0.62	0.17	0.30

Easygoing	0.73	0.55	0.71
Bold	0.8	0.49	0.66
Confident	0.77	0.51	0.67
Curious	0.53	0.17	0.29
Independent	0.58	0.36	0.53
Obedient	0.72	0.48	0.65
Willing to learn	0.69	0.58	0.73
Patient	0.73	0.58	0.74
Calm	0.69	0.62	0.77
Empathic	0.63	0.34	0.51
Predictable	0.63	0.20	0.33
Reliable	0.79	0.44	0.61
Attentive	0.7	0.38	0.55
Focused	0.7	0.38	0.55
Intelligent	0.53	0.19	0.32
Restless	0.78	0.45	0.62
Excitable	0.7	0.70	0.82
Provocative	0.68	0.59	0.74
Stubborn	0.74	0.52	0.69
Distractible	0.71	0.55	0.71
Impulsive	0.65	0.48	0.65
Playful with people	0.51	0.20	0.33
Energetic	0.71	0.45	0.62
Boisterous	0.71	0.51	0.67
Active	0.62	0.48	0.65
Playful alone	0.64	0.59	0.75
Slow	0.77	0.36	0.52
Lazy	0.76	0.43	0.60
Aggressive to dogs (same gender)	0.84	0.60	0.75
Aggressive to dogs (opposite gender)	0.69	0.74	0.85
Dominant	0.77	0.47	0.64
Territorial	0.67	0.55	0.71
Sociable with dogs (same gender)	0.74	0.56	0.72
Submissive	0.73	0.50	0.67
Calming	0.65	0.46	0.63
Human oriented	0.71	0.41	0.58
Sociable with people	0.73	0.37	0.54
Affectionate with people	0.72	0.38	0.55
Attention seeking	0.66	0.45	0.62
Solitary	0.61	0.49	0.66
Playful with dogs	0.7	0.71	0.83
Affectionate with dogs	0.75	0.57	0.73
Sociable with dogs (opposite gender)	0.71	0.43	0.60
Indifferent	0.77	0.56	0.72
Decisive	0.51	0.30	0.46
Persevering	0.4	0.18	0.31
REMOVED ITEMS			
Vocal	0.77	0.52	0.69
Prey driven (chase)	0.69	0.62	0.76
Prey driven (search)	0.8	0.58	0.73
Greedy	0.86	0.62	0.76
Vigilant	0.52	0.49	0.66

Loadings > 0.30 and < -0.30 are in bold.

References

1. Chopik, W.J.; Weaver, J.R. Old dog, new tricks: Age differences in dog personality traits, associations with human personality traits, and links to important outcomes. *J. Res. Pers.* **2019**, *79*, 94–108, doi:10.1016/j.jrp.2019.01.005.
2. Ley, J.M.; Bennett, P.C.; Coleman, G.J. A refinement and validation of the Monash Canine Personality Questionnaire (MCPQ). *Appl. Anim. Behav. Sci.* **2009**, *116*, 220– 227, doi:10.1016/j.applanim.2008.09.009.
3. Wallis, L.J.; Szabó, D.; Kubinyi, E. Cross-Sectional Age Differences in Canine Personality Traits; Influence of Breed, Sex, Previous Trauma, and Dog Obedience Tasks. *Front. Vet. Sci.* **2020**, *6*, 493, doi:10.3389/fvets.2019.00493.
4. Harvey, N.D.; Craigon, P.J.; Blythe, S.A.; England, G.C.W.; Asher, L. Social rearing environment influences dog behavioral development. *J. Vet. Behav. Clin. Appl. Res.* **2016**, *16*, 13–21, doi:10.1016/j.jveb.2016.03.004.
5. Eken Asp, H.; Fikse, W.F.; Nilsson, K.; Strandberg, E. Breed differences in everyday behaviour of dogs. *Appl. Anim. Behav. Sci.* **2015**, *169*, 69–77, doi:10.1016/j.applanim.2015.04.010.
6. Serpell, J.A.; Duffy, D.L. Dog breeds and their behavior. In *Domestic Dog Cognition and Behavior*; Horowitz, A..; Springer, Berlin, Heidelberg, 2014; pp. 31–57 ISBN 9783642539947.
7. Svartberg, K. A comparison of behaviour in test and in everyday life: Evidence of three consistent boldness-related personality traits in dogs. *Appl. Anim. Behav. Sci.* **2005**, *91*, 103–128, doi:10.1016/j.applanim.2004.08.030.
8. Martínez, Á.G.; Santamarina Pernas, G.; Diéguez Casalta, F.J.; Suárez Rey, M.L.; De la Cruz Palomino, L.F. Risk factors associated with behavioral problems in dogs. *J. Vet. Behav. Clin. Appl. Res.* **2011**, *6*, 225–231, doi:10.1016/j.jveb.2011.01.006.
9. Col, R.; Day, C.; Phillips, C.J.C. An epidemiological analysis of dog behavior problems presented to an Australian behavior clinic, with associated risk factors. *J. Vet. Behav. Clin. Appl. Res.* **2016**, *15*, 1–11, doi:10.1016/J.JVEB.2016.07.001.
10. Hsu, Y.; Sun, L. Factors associated with aggressive responses in pet dogs. *Appl. Anim. Behav. Sci.* **2010**, *123*, 108–123, doi:10.1016/j.applanim.2010.01.013.
11. Dinwoodie, I.R.; Dwyer, B.; Zottola, V.; Gleason, D.; Dodman, N.H. Demographics and comorbidity of behavior problems in dogs. *J. Vet. Behav. Clin. Appl. Res.* **2019**, *32*, 62–71, doi:10.1016/j.jveb.2019.04.007.
12. Duffy, D.L.; Hsu, Y.; Serpell, J.A. Breed differences in canine aggression. *Appl. Anim. Behav. Sci.* **2008**, *114*, 441–460, doi:10.1016/j.applanim.2008.04.006.
13. Bamberger, M.; Houpt, K.A. Signalment factors, comorbidity, and trends in behavior diagnoses in dogs: 1,644 cases (1991–2001). *J. Am. Vet. Med. Assoc.* **2006**, *229*, 1591– 1601, doi:10.2460/javma.229.10.1591.
14. Kubinyi, E.; Turcsán, B.; Miklósi, Á. Dog and owner demographic characteristics and dog personality trait associations. *Behav. Processes* **2009**, *81*, 392–401, doi:10.1016/j.beproc.2009.04.004.
15. Starling, M.J.; Branson, N.; Thomson, P.C.; McGreevy, P.D. “Boldness” in the domestic dog differs among breeds and breed groups. *Behav. Processes* **2013**, *97*, 53– 62, doi:10.1016/j.beproc.2013.04.008.
16. Tonoike, A.; Nagasawa, M.; Mogi, K.; Serpell, J.A.; Ohtsuki, H.; Kikusui, T. Comparison of owner-reported behavioral characteristics among genetically clustered breeds of dog (*Canis familiaris*). *Sci. Rep.* **2015**, *5*, 17710, doi:10.1038/srep17710.
17. Storengen, L.M.; Lingaas, F. Noise sensitivity in 17 dog breeds: Prevalence, breed risk and correlation with fear in other situations. *Appl. Anim. Behav. Sci.* **2015**, *171*, 152–160, doi:10.1016/j.applanim.2015.08.020.
18. Blackwell, E.J.; Bradshaw, J.W.S.; Casey, R.A. Fear responses to noises in domestic dogs: Prevalence, risk factors and co-occurrence with other fear related behaviour. *Appl. Anim. Behav. Sci.* **2013**, *145*, 15–25, doi:10.1016/j.applanim.2012.12.004.
19. Dale, A.R.; Walker, J.K.; Farnworth, M.J.; Morrissey, S. V.; Waran, N.K. A survey of owners’ perceptions of fear of fireworks in a sample of dogs and cats in New Zealand. *N. Z. Vet. J.* **2010**, *58*, 286–291, doi:10.1080/00480169.2010.69403.
20. McGreevy, P.D.; Georgevsky, D.; Carrasco, J.; Valenzuela, M.; Duffy, D.L.; Serpell, J.A. Dog behavior co-varies with height, bodyweight and skull shape. *PLoS One* **2013**, *8*, e80529, doi:10.1371/journal.pone.0080529.
21. Bennett, P.C.; Rohlf, V.I. Owner-companion dog interactions: Relationships between demographic variables, potentially problematic behaviours, training engagement and shared activities. *Appl. Anim. Behav. Sci.* **2007**, *102*, 65–84, doi:10.1016/j.applanim.2006.03.009.
22. Casey, R.A.; Loftus, B.; Bolster, C.; Richards, G.J.; Blackwell, E.J. Human directed aggression in domestic dogs (*Canis familiaris*): Occurrence in different contexts and risk factors. *Appl. Anim. Behav. Sci.* **2014**, *152*, 52–63, doi:10.1016/j.applanim.2013.12.003.
23. Flint, H.E.; Coe, J.B.; Serpell, J.A.; Pearl, D.L.; Niel, L. Risk factors associated with stranger-directed aggression in domestic dogs. *Appl. Anim. Behav. Sci.* **2017**, *197*, 45– 54, doi:10.1016/J.APPLANIM.2017.08.007.
24. Hakanen, E.; Mikkola, S.; Salonen, M.; Puurunen, J.; Sulkama, S.; Araujo, C.; Lohi, H. Active and social life associates with lower non-social fear in pet dogs. *Sci. Rep.* **2020**, *10*, 13774, doi:10.1038/s41598-020-70722-7.
25. Vas, J.; Topál, J.; Péch, É.; Miklósi, Á. Measuring attention deficit and activity in dogs: A new application and validation of a human ADHD questionnaire. *Appl. Anim. Behav. Sci.* **2007**, *103*, 105–117, doi:10.1016/j.applanim.2006.03.017.
26. Wright, H.F.; Mills, D.S.; Pollux, P.M.J. Development and validation of a psychometric tool for assessing impulsivity in the domestic dog (Canis familiaris). *Int. J. Comp. Psychol.* **2011**, *24*, 210–225.
27. Lit, L.; Schweitzer, J.B.; Iosif, A.-M.; Oberbauer, A.M. Owner reports of attention, activity, and impulsivity in dogs: a replication study. *Behav. Brain Funct.* **2010**, *6*, 1, doi:10.1186/1744-9081-6-1.
28. Vas, J.; Topál, J.; Péch, É.; Miklósi, Á. Measuring attention deficit and activity in dogs: A new application and validation of a human ADHD questionnaire. *Appl. Anim. Behav. Sci.* **2007**, *103*, 105–117, doi:10.1016/j.applanim.2006.03.017.

Supplementary file: Questionnaire

Table of Contents

Dog's background 155

Health survey 233

Noise phobia 39

Shyness/fearfulness 544

Aggressiveness 633

Surface phobia 69

Separation anxiety 722

Impulsivity/inattention 755

Cognition (perception, learning and memory) 822

Stereotypic behavior 88

Personality survey 109

Breed information (for mixed breed dogs) 1222

Dog's background

Many behaviors are affected not only by genes but also by the dog's experiences, particularly in early life. Please take time to answer the following questions about your dog's history. You may have to contact your dog's breeder to be able to answer some of the questions.

Asterisk indicates mandatory field.

Data protection *
Hereby, I accept that my personal information, the information of my dog and all of the data collected with this questionnaire I have provided is transferred to Canine Genetics research group at the University of Helsinki and used in scientific research. Read the privacy policy of the Canine Genetics Group (only in Finnish).

1. What is the main reason to have this dog? *
    Family member
    Dog hobbies
    Pet
    Working dog (if ticked, Q1.1. is shown)

    1.1. What kind of work does your dog do? *
        Detection dog (e.g. mold, cancer)
        Hunting dog
        Herding dog
        Watch dog/livestock guardian dog
        Service/assistance dog
        Search and rescue dog
        Working dog in police/army/customs force
        Sled dog
        Therapy dog
        Other

2. How did you obtain the dog? *
    The dog was born at my home
    From a breeder
    From previous owner (if ticked, Q2.1 is shown)
    Dog is rescue/stray
    From elsewhere (if ticked, Q2.2. is shown)

    2.1. Why didn't the previous owners keep the dog? *
        allergy or other disease of the family member
        dog had behavioral problems

changed life situation of owners
previous owner died
Some other reason (If ticked Q3.1.1. is shown)
I don't know

2.1.1. What is the other reason? _________________

2.2. Where did obtain your dog? _________________

3. If your dog's breed has different lines (e.g. Border collie, German shepherd, Labrador retriever), what is your dog's line?
Mixed line
Working/field/hunting/herding
Show

4. At what age did your dog enter your household? *
under 4 months of age (if ticked, Q4.1-Q4.9 are shown)
4 - 6 months of age
6 months - 1 year of age
1 - 1,5 years of age
1,5 - 2 years of age
2 - 3 years of age
3 - 6 years of age
over 6 years of age

4.1. At what age was your dog separated from its mother or surrogate mother? *
Under 4 months of age
5 weeks of age
6 weeks of age
7 weeks of age
8 weeks of age
9 weeks of age
10-12 weeks of age
Over 12 weeks of age
Dogs still lives in the same household with the dam
I don't know

4.2. How did the mother of your dog take care of the puppies? *
Mother took extremely good care of the puppies and spent a lot of time with them
Mother took good care of the puppies
Mother took relatively good care of the puppies, but sometimes she had to be encouraged/told to go spend time with the puppies

At the beginning the mother spent time with the puppies, but later started to avoid being with them
Mother did not want to spend time with the puppies but nursed them
Mother did not take care of the puppies; the surrogate mother or bottle-feeding was needed
Other way (If ticked, Q4.2.1. is shown)
I don't know

4.2.1. Please describe the mother's behaviour: ________________

The socialization period: between 7 weeks and 4 months of age, how often did your dog..

4.3. Meet unfamiliar men? *
Very often (several times a day)
Often (twice a week-once a day)
Sometimes (twice a month-twice a week)
Rarely (1 - 4 times during puppyhood)
Never

4.4. Meet unfamiliar women? *
Very often (several times a day)
Often (twice a week-once a day)
Sometimes (twice a month-twice a week)
Rarely (1 - 4 times during puppyhood)
Never

4.5. Meet unfamiliar children? *
Very often (several times a day)
Often (twice a week-once a day)
Sometimes (twice a month - twice a week)
Rarely (1 - 4 times during puppyhood)
Never

4.6. Meet unfamiliar dogs? *
very often (several times a day)
often (twice a week-once a day)
sometimes (twice a month-twice a week)
rarely (1- 4 times during puppyhood)
never

4.7. Visit city center? *
Very often (several times a day)
Often (twice a week-once a day)
Sometimes (twice a month-twice a week)

Rarely (1 - 4 times during puppyhood)
Never

4.8. Travel by car? *
Very often (several times a day)
Often (twice a week-once a day)
Sometimes (twice a month-twice a week)
Rarely (1 - 4 times during puppyhood)
Never

4.9. Travel by public transportation? *
Very often (several times a day)
Often (twice a week-once a day)
Sometimes (twice a month-twice a week)
Rarely (1 - 4 times during puppyhood)
Never

5. In what kind of environment do you live? *
In a city center
In a city/town, but outside the center
In a rural area

6. In what kind of building do you live? *
In an apartment
In a row house/semi-detached house
In (detached) house
Other

7. Does the dog have outdoor access daily or almost every day (not including walking with the dog)? *
No
Yes, in a kennel
Yes, tethered in the backyard
Yes, freely (not tethered) in the backyard

8. Your dog lives *
indoors
outdoors

9. The number of adults in your family * ____________________

10. The number of children in your family *___________________

11. Were there other dogs in household when you got this dog to family? *

yes

no

12. Are there other dogs currently living with this dog? * (If the answer is "yes", Q 12.1. is shown)

yes

no

12.1. How many other dogs? _________

13. How many dogs have you had (living in **your household**)? *

This is my first dog

This is my second dog

This is my third to fifth dog

This is my sixth dog or over

14. How many times does your dog get exercise in a typical day? *

three times or more

twice a day

once a day

dog is outside all the time

something else (if ticked, Q14.1. is shown)

14.1. Please specify: ___________

15. During a typical day, how many hours/minutes do you walk your dog? *

three hours or more

2 - 3 hours

1 - 2 hours

30 min - 1 hour

less than 30 min

16. During the daily walks the dog is *

on a leash during the whole walk

on a leash for part of the walk and allowed to run free for part of the walk

mostly allowed to run free during the walks

17. Do you have any hobbies with your dog e.g. agility, obedience **not including** daily walks? * (If answer is "yes", Q17.1-Q17.3 are shown.)

yes

no

17.1. What hobbies do you have? You can choose multiple hobbies. *

dog shows
obedience training
rally obedience
agility
nose work/scent training
tracking, protection etc.
search and rescue
water rescue
hunting trials
hunting in practice
skijoring
canicross
herding
something else

17.2. How often do you do these activities with your dog **at home** (hobbies altogether)? In this we mean e.g. agilility or obedience, not daily exercise. *

Never
A few times a year
1 - 3 times a month
1 - 2 times a week
3 - 4 times a week
Almost every day
Several times a day

17.3. How often do you do these activities with your dog **outside of your home** (e.g. with an instructor)? In this we mean e.g. agilility or obedience, not daily exercise. *

Never
A few times a year
1 - 3 times a month
1 - 2 times a week
3 - 4 times a week
Almost every day

18. What kind of food does your dog mainly eat? *

Raw food/BARF
Partially raw food, partially dog food
Homemade food made for the dog
Table scraps
Dog food purchased from a pet store
Dog food purchased from a grocery store/supermarket

Something else (if ticked, Q18.1. is shown)

18.1. Please specify what you feed to your dog: ________________________

19. Does your dog get any supplements? * (If answer is "Yes", Q19.1-Q19.4. are shown.)
Yes
No

19.1. How often do your dog get supplements? *
daily
weekly
occasionally

19.2. What supplements do you give your dog? You can choose multiple options. *
Vitamins and/or minerals
Joint supplement
Vegetable oil
Fish oil
Antioxidants (e.g. vitamin C and vitamin E)
Natural health products (besides those mentioned above)
Something else (if ticked, Q19.2.1. is shown)

19.2.1. Please specify, what other supplements you give: __________________

19.3. When did you start to give supplements to your dog?
When the dog was a puppy (0 -1 years)
When the dog was an adult (1 - 6 years)
When the dog was a senior (7 years or older)

19.4. Why did you start to give supplements? __________________

20. How much time does your dog spend **alone** (without people present) during a typical working day? *
0 hours
0 - 1 hours
1 - 3 hours
3 - 6 hours
6 - 8 hours
8 - 9 hours
9 - 10 hours
10 hours or more

21. What kind of experience do you have of dogs? Choose all the options that describe your dog experiences. *

Family dog(s) during childhood
Dog-related hobby during childhood/teenage years (e.g. junior handler, dog sitter)
Family dogs in teenage years
First dog during adulthood. No dogs before that in the family.

22. How many years have you or your household had dogs? ____________

23. Are you a dog breeder? * (If answer is ″Yes″, Q23.1.-Q23.3 are shown.)
No
Yes

23.1. How many litters have you raised? ___________________

23.2. What year was your first litter born in? ___________________

23.3. What year was your last litter born in? ___________________

24. Do you work with animals/animal industry? * (If answer is ″Yes″, Q24.1. is shown.)
No
Yes

24.1. What is your profession? *
Veterinarian
Veterinary technicians
Pet shop staff
Animal trainer
Animal physical therapatist
Researcher
Other (If ticked, Q22.1.1 is shown)

24.1.1. What other profession? ________________

Health survey

Health problems, including multiple diseases and injuries can affect the behavior of your dog. Sometimes the changes in the behavior are temporary, but some health issues, for example blindness or joint pains, can change the behavior permanently. Please answer the questions in this section carefully, so that we can take the possible health problems into account when studying dog behavior.

Asterisk indicates mandatory field. You can fill this survey again anytime and it will show in available surveys.

Data protection *
Hereby, I accept that my personal information, the information of my dog and all of the data collected with this questionnaire I have provided is transferred to Canine Genetics research group at the University of Helsinki and used in scientific research. Read the privacy policy of the Canine Genetics Group (only in Finnish).

1. Is your dog deceased? * (If answer is “Yes”, Q1.1. is shown.)
   Yes
   No

   1.1. When did your dog pass away? * ____________

2. What is your dog heights at withers (in cm)? _________

3. How much does your dog weigh (in kilos)? ____________

4. How would you describe the body condition of your dog? *
   Severely underweight
   Somewhat underweight
   Ideal weight
   Somewhat overweight
   Severely overweight
   I don't know

5. Do you brush your dog’s teeth? *
   Yes, daily
   Yes, couple times in a week
   Yes, once a week
   Yes, less often than once a week
   Never

6. Does your dog have a congenital defect (birth defect)? * (If answer is ”Yes”, Q6.1. is shown.)
   Yes
   No

6.1. What kind of congenital defect does your dog have? ____________

7. Is your dog on medication at the moment? * (If answer is "Yes", Q7.1 and Q7.2. are shown.)
Yes
No

7.1. What medicine do you give to your dog? _________

7.2. What health problem the medicine is used for? ___________

8. Do you use natural products, e.g Adaptil or Zylkene? * (If answer is "Yes", Q8.1 and Q8.2. are shown.)
Yes
No

8.1. What natural products do you use? _____________

8.2. For what problem are these products used for? ____________

9. When was the last time your dog visited to a veterinarian? *
Less than 6 months ago
6 - 12 months ago
1 - 2 years ago
2 - 5 years ago
Over 5 years ago
Never

10. Are your dog's vaccinations up to date? *
Yes
No, my dog has been vaccinated but the vaccinations are overdue
No, my dog has been vaccinated only when it was a puppy
No, my dog has never been vaccinated
I don't know

11. When was the last time your dog was dewormed or her/his fecal sample brought to a veterinary clinic? *
Less than 6 months ago
6 - 12 months ago
1 - 2 years ago
2 - 5 years ago
Over 5 years ago
Never

Does your dog have any of the following conditions? Choose all conditions that your dog has/has had.

12. Skin, glands and fur
 Itching (if ticked, Q12.1. is shown)
 Recurrent skin infections (if ticked, Q12.2. is shown)
 Fungal infection (if ticked, Q12.3. is shown)
 Furunculosis (if ticked, Q12.4. is shown)
 Anal gland problem (if ticked, Q12.5. is shown)
 Atopy (if ticked, Q12.6. – Q12.8. are shown)
 Other problems of the skin, glands or fur (e.g. severe dandruff) (if ticked, Q12.9. and Q12.10. are shown)

12.1. Does any disease or other reason cause the itching? You can choose multiple answers.
 No
 Allergy
 Atopy
 Joint pain or other pain
 Fungal infection
 Stress
 Other reason (if ticked, Q12.1.1. is shown)

12.1.1. What other reason causes the itching? _________

12.2. How often does your dog have skin infections?
 More often than once a month
 Less than monthly

12.3. When was the last time your dog had a fungal infection?
 At the moment
 Less than half a year ago
 More than half a year ago

12.4. When was furunculosis noticed the last time?
 At the moment
 Less than half a year ago
 More than half a year ago

12.5. When was the last time your dog had anal gland problems?
 At the moment
 Less than half a year ago
 More than half a year ago

12.6. When was the last time your dog had atopy symptoms?
At the moment
Less than half a year ago
More than half a year ago

12.7. Does your dog's atopy cause symptoms periodically or all the time?
From time to time
Continually

12.8. How severe the atopy symptoms are?
Needs medication continually
Needs medication time to time
Doesn't need medication

12.9. What other skin, gland or fur problem does your dog have?____________

12.10. When was the last time your dog had this problem/disease?
At the moment
Less than half a year ago
More than half a year ago

13. Eyes
Blind (if ticked, Q13.1. and Q13.2. are shown)
Weakened eyesight (if ticked, Q13.3. is shown)
Eye infection (if ticked, Q13.4. is shown)
Kataract (if ticked, Q13.5. is shown)
Other eye-related disease/problem (if ticked, Q13.6 and Q13.7. are shown)

13.1. When was your dog's blindness noticed? Select "I don't know" if your dog was already blind when you acquired her/him.
My dog has been blind from the birth
Less than 3 months ago
3 - 6 months ago
6 - 12 months ago
More than a year ago
I don't know

13.2. How was blindness diagnosed?
By a veterinarian
By myself, based on the behavior of my dog

13.3. When was your dog’s weakened eyesight noticed? Select “I don’t know” if your dog's eyesight was already weakened when you acquired it.

Less than 3 months ago
3 - 6 months ago
6 - 12 months ago
More than a year ago
I don't know

13.4. When was the last time your dog had an eye infection?

At the moment
Less than half a year ago
More than half a year ago

13.5. When was kataract diagnosed?

Less than 3 months ago
3 - 6 months ago
6 - 12 months ago
More than a year ago
I don't know

13.6. What other eye-related disease/problem does your dog have? _________________

13.7. When was the last time your dog had this other eye disease/problem?

At the moment
Less than half a year ago
More than half a year ago

14. Ears

Deaf (if ticked, Q14.1. - Q14.3. are shown)
Weakened hearing (if ticked, Q14.4. is shown)
Ear infection (if ticked, Q14.5. is shown)
Other ear-related disease/problem (if ticked, Q14.6. and Q14.7. are shown)

14.1. Is your dog deaf from both ears?

Yes
Just from one

14.2. When did your dog become deaf? Select “I don’t know” if your dog was already deaf when you acquired her/him.

My dog has been deaf from the birth
Less than 3 months ago
3 - 6 months ago

6 - 12 months ago
More than a year ago
I don’t know

14.3. How was deafness diagnosed?
By a veterinarian, using BAER
By a veterinarian, using some other method
By myself, based on the behavior of my dog

14.4. When was weakened hearing noticed?
Less than a month ago
3 - 6 months ago
6 - 12 months ago
More than one year ago

14.5. When was the last time your dog had an ear infection?
At the moment
Less than half a year ago
More than half a year ago

14.6. What other ear-related disease/problem does your dog have? _________

14.7. When was the last time your dog had this other ear disease/problem?
At the moment
Less than half a year ago
More than half a year ago

15. Mouth and teeth
Dental calculus (tartar) (if ticked, Q15.1. and Q15.2. are shown)
Gingivitis (inflammation of gum tissue) (if ticked, Q15.3. is shown)
Periodontitis (if ticked, Q15.4. is shown)
Missing/removed teeth (if ticked, Q15.5. is shown)
Malocclusion (if ticked, Q15.6. is shown)
Other mouth or teeth-related disease/problem (if ticked, Q15.7. and Q15.8. are shown)

15.1. When was the last time your dog had dental calculus?
At the moment
Less than half a year ago
More than half a year ago

15.2. How much dental calculus does your dog have?
A little

Moderately
A lot

15.3. When was the last time your dog had gingivitis?
At the moment
Less than half a year ago
More than half a year ago

15.4. When was the last time your dog had periodontitis?
At the moment
Less than half a year ago
More than half a year ago

15.5. How many teeth are missing?
Only a few
About half
All or almost all

15.6. Which malocclusion does your dog have?
Underbite
Overbite
Crossbite
Level bite
Other

15.7. What other mouth or teeth-related disease/problem does your dog have? _______

15.8. When was the last time your dog had this other mouth/teeth disease/problem?
At the moment
Less than half a year ago
More than half a year ago

16. Kidneys and urinary tract
Urinary tract infection (if ticked, Q16.1. is shown)
Bladder stones (urine crystals, uroliths) (if ticked, Q16.2. is shown)
Kidney failure (if ticked, Q16.3. - Q16.5. are shown)
Other kidney or urinary tract related disease/problem (if ticked, Q16.6. and Q16.7. are shown)

16.1. When was the last time your dog had a urinary tract infection?
At the moment
Less than half a year ago
More than half a year ago

16.2. When was the last time your dog had bladder stones?
At the moment
Less than half a year ago
More than half a year ago

16.3. Is your dog's kidney failure acute or chronic?
Acute
Chronic

16.4. When was the kidney failure diagnosed?
Less than half a year ago
More than half a year ago

16.5. When was the last time your dog had symptoms of kidney failure?
At the moment
Less than half a year ago
More than half a year ago

16.6. What other kidney or urinary tract related disease/problem does your dog have? ________

16.7. When was the last time your dog had this other kidney or urinary tract disease/problem?
At the moment
Less than half a year ago
More than half a year ago

17. Liver and pancreas
Chronic liver disease (if ticked, Q17.1. is shown)
Exocrine Pancreatic Insufficiency, EPI (if ticked, Q17.2. is shown)
Pancreatitis (if ticked, Q17.3. is shown)
Other liver or pancreas related disease or problem (if ticked, Q17.4. and Q17.5. are shown)

17.1. When was the last time your dog had symptoms of chronic liver disease?
At the moment
Less than half a year ago
More than a half year ago

17.2. Does EPI cause any symptoms (despite ongoing treatment)?
Yes
No
I don't know

17.3. Is your dog's pancreatitis acute or chronic?

Acute
Chronic

17.4. What liver or pancreas related disease/problem does your dog have? ____________

17.5. When was the last time your dog had this liver or pancreas related disease/problem?
At the moment
Less than half a year ago
More than half a year ago

18. Heart, blood and blood vessels
DCM (dilated cardiomyopathy) (if ticked, Q18.1. is shown)
Heart murmur (if ticked, Q18.2. is shown)
Other heart, blood or blood vessel related disease/problem (if ticked, Q18.3. and Q18.4. are shown)

18.1. When was DCM diagnosed?
Less than half a year ago
More than half a year ago
What symptoms of DCM does your dog have?

18.2. Does heart murmur cause symptoms to your dog?
Yes
No

18.3. What other heart, blood or blood vessel related disease/problem does your dog have? _______

18.4. When was the last time your dog had this other heart, blood or blood vessel disease/problem?
At the moment
Less than half a year ago
More than half a year ago

19. Muscular and skeletal system
Osteoarthritis or other joint pain (if ticked, Q19.1. is shown)
Osteochondrosis (if ticked, Q19.2. is shown)
Hip dysplasia (if ticked, Q19.3. is shown)
Patellar dislocation (if ticked, Q19.4. is shown)
Spondylosis (if ticked, Q19.5. is shown)
Amputation (if ticked, Q19.6. and Q19.7. are shown)
Injury caused by traumatic accident (if ticked, Q19.8. is shown)
Other muscular or skeletal system related disease/problem (if ticked, Q19.9. and Q19.10. are shown)

19.1. Does osteoarthritis/other joint pain cause behavioral changes in your dog?

No
Mild, there are small changes in the mood or movement
Moderate, my dog may seems painful when lying down or getting up
Severe, my dog does not want to e.g. run, jump or play at all

19.2. Does osteochondrosis cause symptoms to your dog?
No
Mild symptoms
Severe symptoms
19.3. Does hip dysplasia cause symptoms to your dog?
No
Mild symptoms
Strong symptoms

19.4. Does patellar dislocation cause symptoms to your dog?
No
Mild symptoms
Strong symptoms

19.5. How severe is your dog's spondylosis?
Only bony spurs
One or more bony bridges

19.6. What was amputated?
One front leg
Both front legs
One hind leg
Both hind legs
Whole tail
Tip of the tail or part of the tail
A toe
Several toes
Something else

19.7. When did your dog have amputation surgery?
Less than half a year ago
Half a year ago - year ago
More than a year ago
I don’t know

19.8. What kind of injury does your dog have? How does it affect the behavior of your dog? _________

19.9. What other muscular or skeletal system related disease/problem does your dog have? ________

19.10. When was the last time your dog had this other muscular or skeletal system related disease/problem?
At the moment
Less than half a year ago
More than half a year ago

20. Digestive system
Heartburn (if ticked, Q20.1. is shown)
Repetitive diarrhea (if ticked, Q20.2. is shown)
Gastric dilatation-volvulus (GDV, bloat) (if ticked, Q20.3. is shown)
Other digestion related disease/problem (if ticked, Q20.4 and Q20.5. are shown)

20.1. How often does your dog have heartburn?
Daily
Weekly
Less than weekly

20.2. How often does your dog have diarrhea?
Daily
Several times a week
Once a week
Less than once a week

20.3. When was the last time your dog had gastric diltation volvulus (GDV)?
Less than a week ago
A week - a month ago
A month - 6 months ago
Over 6 months ago

20.4. What other digestion related disease/problem does your dog have? ________

20.5. When was the last time your dog had this digestion related disease/problem?
At the moment
Less than half a year ago
More than half a year ago

21. Respiratory system
Asthma (if ticked, Q21.1. and Q21.2. are shown)
Respiratory infection (if ticked, Q21.3. is shown)
Other respiratory system related disease/problem (if ticked, Q21.4. and Q21.5. are shown)

21.1. What symptoms does asthma cause to your dog? __________

21.2. Does your dog have asthma symptoms?
Daily
Weekly
Less than weekly

21.3. When was the last time your dog had a respiratory infection?
At the moment
Less than half a year ago
More than half a year ago

21.4. What other respiratory system related disease/problem does your dog have? _______

21.5. When was the last time your dog had this other respiratory system related disease/problem?
At the moment
Less than half a year ago
More than half a year ago

22. Nervous system
Epilepsy (if ticked, Q22.1. and Q22.2. are shown)
Ataxia (if ticked, Q22.3. and Q22.3. are shown)
Other nervous system related disease/problem (if ticked, Q22.4. and Q22.5. are shown)

22.1. When was the last time your dog had an epileptic seizure?
Less than a week ago
One week - a month ago
A month - 6 months ago
Over 6 months ago

22.2. How often does your dog get epileptic seizures?
Daily
Weekly
Couple times in a month
Monthly
Less often than monthly

22.3. How does ataxia affect your dog's life? __________

22.4. When was the last time ataxia was noticed?
At the moment
Less than half a year ago

More than half a year ago

22.5. What other nervous system related disease/problem does your dog have? _________

22.6. When was the last time this other nervous system related disease/problem caused problems to your dog?
At the moment
Less than half a year ago
More than half a year ago

27. Reproductive organs
Uterine infection (pyometra) (if ticked, Q27.1. is shown)
Prostatitis (prostate infection) (if ticked, Q27.2. is shown)
Undescended testicle
Other reproductive disease or problem (if ticked, Q27.3. and Q27.4. are shown)

27.1. When was your dog's uterine infection noticed?
Less than a month ago
A month - 6 months ago
More than 6 months ago

27.2. When was prostatitis noticed?
More than 6 months ago
Less than a month ago
A month - 6 months ago

27.3. What other reproductive disease or problem does your dog have? _____________

27.4. When was this reproductive disease or problem noticed the last time?
At the moment
Less than half a year ago
More than half a year ago

28. Hormones and metabolism
Hypothyroidism (if ticked, Q28.1. and Q28.2. are shown)
Diabetes mellitus type 2 (if ticked, Q28.3. and Q28.4. are shown)
Other hormone or metabolism related disease/problem (if ticked, Q28.5. and Q28.6 are shown)

28.1. When was your dog’s hyporthyroidism diagnosed?
Less than half a year ago
More than half a year ago

28.2. When was the last time hyperthyroidism caused symptoms to your dog?

At the moment
Less than half a year ago
More than half a year ago

28.3. When was diabetes diagnosed?
Less than a month ago
Less than six months ago
Over six months ago

28.4. If your dog currently has symptoms of diabetes, please describe them. _________

28.5. What hormone or metabolism related disease/problem does your dog have? ________

28.6. When was the last time this hormone or metabolism related disease/problem was noticed?
At the moment
Less than half a year ago
More than half a year ago

29. Allergies and autoimmune diseases
SLO (symmetrical lupoid onychodystrophy) (if ticked, Q29.1. is shown)
Food allergy (if ticked, Q29.2. is shown)
Other allergy or autoimmune disease (if ticked, Q29.3. and Q29.4. are shown)

29.1. Does SLO affect the behavior of your dog? ___________

29.2. What symptoms does food allergy cause to your dog? You can choose multiple answers.
Skin symptoms
Ear redness/itching/infection
Gastrointestinal symptoms
Some other symptoms ((if ticked, Q29.2.1 is shown)

29.2.1. What other symptoms does food allergy cause to your dog? __________

29.3. What allergy or autoimmune disease does your dog have? __________

29.4. When was this allergy or autoimmunity disease diagnosed?
At the moment
Less than half a year ago
More than half a year ago

30. Cancers and tumors
Benign tumor (e.g. lipoma) (if ticked, Q30.1. is shown)

Malignant tumor (cancer) (if ticked, Q30.2. and Q30.3. are shown)
Undefined tumor (if ticked, Q30.4. is shown)

30.1. Does the tumor cause symptoms? What symptoms? ____________

30.2. What cancer does/did your dog have?
Lymphoma
Mammary tumor
Bone tumor
Skin cancer
Other ((if ticked, Q30.2.1. is shown)

30.2.1. Please, describe what cancer your dog has. _________

30.3. Was the cancer removed?
Yes
No

30.4. What tumor does/did your dog have? __________

31. Parasites and protozoans
Internal parasites/intestinal parasites (e.g. tapeworms, roundworms) (if ticked, Q31.1. is shown)
External parasites (e.g.*Demodex canis*, ticks, mange) (if ticked, Q31.2. and Q31.3. are shown)
Protozoans (e.g. giardia, toxoplasma gondii) (if ticked, Q31.4. is shown)

31.1. When were internal parasites noticed the last time?
Less than a week ago
Less than half a year ago
More than half a year ago

31.2. When were external parasites noticed the last time?
At the moment
Less than half a year ago
More than half a year ago

31.3. What external parasites the dog has had within the last month?
Demodex canis
Lice
Manges
Ticks
Cheyletiella mites
Canine nasal mites

Ear mites
Fleas
Other

31.4. When were protozoans noticed the last time?
Less than a week ago
Less than half a year ago
More than half a year ago

32. Other diseases and health problems
Kennel cough (canine infectious tracheobronchitis) (if ticked, Q32.1. is shown)
Lyme disease (if ticked, Q32.2. is shown)
Pain (if ticked, Q32.3. and Q32.4 are shown)

32.1. When did the symptoms of the last kennel cough end?
My dog still has symptoms / less than a week ago
Less than a month ago
More than a month ago

32.2. What symptoms does lyme disease cause to your dog?
Neurological symptoms
Limping
Fever
Stiffness / difficulties in movement
Neurological pain
Something else

32.3. Where does your dog have pain and what symptoms does it cause? ___________

32.4. In your opinion, how severe are the pain symptoms?
Mild
Moderate
Severe

33. If you did not find your dog's disease or health problem, describe it below.
____

Noise phobia

If you answer "I don't know" to a question, the report of that part of the questionnaire will be empty.

Asterisk indicates mandatory field.

Data protection *
Hereby, I accept that my personal information, the information of my dog and all of the data collected with this questionnaire I have provided is transferred to Canine Genetics research group at the University of Helsinki and used in scientific research. Read the privacy policy of the Canine Genetics Group (only in Finnish).

1. **Has your dog ever heard thunder?*** (If answer is "Yes", Q1.1.-1.16. are shown.)
    Yes
    No

    1.1. How often does your dog hear thunder? *
        Less frequently than once a year
        Couple of times in a year
        Several times in a year

    Please note how often the statement is true for your dog, when she/he hears thunder

    1.2. My dog escapes *
        Never
        Rarely
        Sometimes
        Often
        Always or almost always
        I don't know

    1.3. My dog pants *
        Never
        Rarely
        Sometimes
        Often
        Always or almost always
        I don't know

    1.4. My dog hides (e.g. under the bed) *
        Never
        Rarely

Sometimes
Often
Always or almost always
I don't know

1.5. My dog trembles *
Never
Rarely
Sometimes
Often
Always or almost always
I don't know

1.6. My dog paces *
Never
Rarely
Sometimes
Often
Always or almost always
I don't know

1.7. My dog's tail is low/between legs *
Never
Rarely
Sometimes
Often
Always or almost always
I don't know

1.8. My dog freezes *
Never
Rarely
Sometimes
Often
Always or almost always
I don't know

1.9. My dog barks or growls *
Never
Rarely
Sometimes
Often

Always or almost always
I don't know

1.10. My dog vocalizes in any other way *
Never
Rarely
Sometimes
Often
Always or almost always
I don't know

1.11. My dog salivates *
Never
Rarely
Sometimes
Often
Always or almost always
I don't know

1.12. My dog stays close to the owner *
Never
Rarely
Sometimes
Often
always or almost always
I don't know

1.13. My dog is indifferent to the sound *
Never
Rarely
Sometimes
Often
Always or almost always
I don't know

1.14. My dog notices the sound, but continues to do what she/he was doing *
Never
Rarely
Sometimes
Often
Always or almost always
I don't know

1.15. In your opinion, is your dog afraid of thunder? * (If answer is "Yes", Q1.15.1. and Q1.15.2. are shown.)
Yes
No

1.15.1. When was the fearful reaction towards thunder first observed? *
At 1.5 - 2 years of age
Before 3 months of age
At 3 months - 6 months of age
At 6 months - 1 year of age
At 1 year - 1.5 year of age
At 2 - 3 years of age
At 3 - 6 years of age
Older than 6 years of age
My dog was already fearful when I got him/her

1.15.2. How long does your dog's fearful reaction last after the thunder has stopped? The dog returns to normal... *
Right after
In a few minutes
In 15 mins - 1 hour
In 1 - 5 hours
In more than 5 hours
I don't know

1.16. Has there been any changes towards thunder within the last six months? *
The dog's reaction has stayed the same
The dog has become fearful towards thunder (wasn't before)
The fear of thunder has increased
The fear of thunder has decreased
The dog is no longer afraid of thunder at all

2. Has your dog ever heard fireworks? * (If answer is "Yes", Q2.1.-2.16. are shown.)
Yes
No

2.1. How often does your dog hear fireworks? *
Less frequently than once a year
Once a year
Several times in year

Please note how often the statement is true for your dog, when she/he hears fireworks.

2.2. My dog escapes *
Never
Rarely
Sometimes
Often
Always or almost always
I don't know

2.3. My dog pants *
Never
Rarely
Sometimes
Often
Always or almost always
I don't know

2.4. My dog hides (e.g. under the bed) *
Never
Rarely
Sometimes
Often
Always or almost always
I don't know

2.5. My dog trembles *
Never
Rarely
Sometimes
Often
Always or almost always
I don't know

2.6. My dog paces *
Never
Rarely
Sometimes
Often
Always or almost always
I don't know

2.7. My dog's tail is low/between legs *
Never

Rarely
Sometimes
Often
Always or almost always
I don't know

2.8. My dog freezes *
Never
Rarely
Sometimes
Often
Always or almost always
I don't know

2.9. My dog barks or growls *
Never
Rarely
Sometimes
Often
Always or almost always
I don't know

2.10. My dog vocalizes in any other way *
Never
Rarely
Sometimes
Often
Always or almost always
I don't know

2.11. My dog salivates *
Never
Rarely
Sometimes
Often
Always or almost always
I don't know

2.12. My dog stays near the owner *
Never
Rarely
Sometimes

Often
Always or almost always
I don't know

2.13. My dog is indifferent to the sound *
Never
Rarely
Sometimes
Often
Always or almost
I don't know

2.14. My dog notices the sound but continues to do what she/he was doing *
Never
Rarely
Sometimes
Often
Always or almost always
I don't know

2.15. In your opinion, is your dog afraid of fireworks? * (If answer is "Yes", Q2.15.1. and Q2.15.2)
Yes
No

2.15.1. When was the fearful reaction towards fireworks first observed? *
At 1.5 - 2 years of age
At 2 - 3 years of age
At 3 - 6 years of age
Older than 6 years of age
My dog was already fearful when I got him/her
At 1 year - 1.5 year of age
At 6 months - 1 year of age
At 3 months - 6 months of age
Before 3 months of age

2.15.2. How long does the dog's fearful reaction last after the fireworks have stopped? The dog returns to normal... *
Right after
In a few minutes
In 15 mins - 1 hour
In 1 - 5 hours
In more than 5 hours

I don't know

2.16. Has there been any changes towards fireworks within the last six months? *

The dog's reaction has stayed the same

The dog has become fearful of fireworks (wasn't before)

The fear of fireworks has increased

The fear of fireworks has decreased

The dog is no longer fearful of fireworks at all

3. Has your dog ever heard a gunshot? * (If answer is ”Yes”, Q3.1.-3.16. are shown.)

Yes

No

3.1. How often does the dog hear gunshots? *

Once or couple of times during the lifetime

About once a year

Less than 10 times in a year

Above 10 times in year

Please note how often the statement is true for your dog, when she/he hears gunshots.

3.2. My dog escapes *

Never

I don't know

Always or almost always

Often

Sometimes

Rarely

3.3. My dog pants *

Never

Rarely

Sometimes

Often

Always or almost always

I don't know

3.4. My dog hides (e.g. under the bed) *

Never

Rarely

Sometimes

Often

Always or almost always
I don't know

3.5. My dog trembles *
Never
Rarely
Sometimes
Often
Always or almost always
I don't know

3.6. My dog paces *
Never
Rarely
Sometimes
Often
Always or almost always
I don't know

3.7. My dog's tail is low/between legs *
Never
Rarely
Sometimes
Often
Always or almost always
I don't know

3.8. My dog freezes *
Never
Rarely
Sometimes
Often
Always or almost always
I don't know

3.9. My dog barks or growls *
Never
Rarely
Sometimes
Often
Always or almost always
I don't know

3.10. My dog vocalizes in any other way *
Never
Rarely
Sometimes
Often
Always or almost always
I don't know

3.11. My dog salivates *
Never
Rarely
Sometimes
Often
Always or almost always
I don't know

3.12. My dog stays near the owner *
Never
Rarely
Sometimes
Often
Always or almost always
I don't know

3.13. My dog is indifferent to the sound *
Never
Rarely
Sometimes
Often
Always or almost always
I don't know

3.14. My dog notices the sound but continues to do what she/he was doing *
Never
Rarely
Sometimes
Often
Always or always almost
I don't now

3.15. In your opinion, is your dog afraid of gunshots? * (If answer is ″Yes″, Q3.15.1 and Q3.15.2. are shown.)
No

Yes

3.15.1. When was the fearful reaction towards gunshots first observed? *

At 6 months - 1 year of age
Before 3 months of age
At 3 - 6 years of age
At 2 - 3 years of age
At 3 months - 6 months of age
At 1 year - 1.5 year of age
At 1.5 - 2 years of age
My dog was already fearful when I got him/her
Older than 6 years of age

3.15.2. How long does the dog's fearful reaction last after the gunshots have stopped? The dog returns to normal... *

Right after
In a few minutes
In 15 mins - 1 hour
In 1 - 5 hours
In more than 5 hours
I don't know

3.16. Has there been any changes towards gunshots within the last six months? *

The dog's reaction has stayed the same
The dog has become fearful of gunshots (wasn't before)
The fear of gunshots has increased
The fear of gunshots has decreased
The dog is no longer fearful of gunshots at all

4. Does your dog react to other sounds? Please, choose all the matching options *

Sudden noise outside (e.g. explosives, bangs)
Long-lasting noise inside (e.g. hair dryer, mixer, drill driver)
Sudden noise inside (e.g. fire alarm, dropping some item)
Sounds of vehicles
Siren (alarm)
Vacuum cleaner
My dog does not react to any sounds
Something else (If ticked, Q4.1. is shown)

4.1. Answer how your dog reacts when dog hears other noises. Please, specify what other noises your dog reacts to. ________________

Please note how often the statement is true for your dog, when she/he hears these sounds

5. My dog escapes *

Never
Rarely
Sometimes
Often
Always or almost always
I don't know

6. My dog pants *

Never
Rarely
Sometimes
Often
Always or almost always
I don't know

7. My dog hides (e.g. under the bed) *

Never
Rarely
Sometimes
Often
Always or almost always
I don't know

8. My dog trembles *

Never
Rarely
Sometimes
Often
Always or almost always
I don't know

9. My dog paces *

Never
Rarely
Sometimes
Often
Always or almost always
I don't know

10. My dog's tail low/between legs *
Never
Rarely
Sometimes
Often
Always or almost always
I don't know

11. My dog freezes *
Never
Rarely
Sometimes
Often
Always or almost always
I don't know

12. My dog barks or growls *
Never
Rarely
Sometimes
Often
Always or almost always
I don't know

13. My dog vocalizes in any other way *
Never
Rarely
Sometimes
Often
Always or almost always
I don't know

14. My dog salivates *
Never
Rarely
Sometimes
Often
Always or almost always
I don't know

15. My dog stays near the owner *
Never

Rarely
Sometimes
Often
Always or almost always
I don't know

16. My dog is indifferent to sound *
Never
Rarely
Sometimes
Often
Always or almost always
I don't know

17. My dog notices the sound but continues to do what she/he was doing *
Never
Rarely
Sometimes
Often
Always or almost always
I don't know

18. In your opinion, is your dog afraid of these sounds? * (If answer is ″Yes″, Q18.1. and Q18.2. are shown.)
Yes
No

18.1. When was the fearful reaction towards other noises first observed? *
Before 3 months of age
At 3 months - 6 months of age
At 6 months - 1 year of age
At 1 year - 1.5 year of age
At 1.5 - 2 years of age
At 2 - 3 years of age
At 3 - 6 years of age
Older than 6 years of age
My dog was already fearful when I got him/her

18.2. How long does the dog's fearful reaction last after the sound has stopped? The dog returns to normal... *
Right after
In a few minutes
In 15 mins - 1 hour
In 1 - 5 hours

In more than 5 hours
I don't know

19. Has there been any changes towards other noises within the last six months? *

The dog's reaction has stayed the same
The dog has become fearful of other noises (wasn't before)
The fear of other noises has increased
The fear of other noises has decreased
The dog is no longer fearful of other noises at all

20. Has your dog ever been treated for noise phobia or desensitized? You can choose multiple options. *

I have not treated or desensitized my dog
Medications
Natural products
Desensitization, e.g. playing recordings of the sound that causes fear
Thundershirt or handmade similar product
Other (If ticked, Q201. is shown.)

20.1. Please specify__________

21. Does your dog have some other symptoms of noise phobia? Describe them. _____________

22. If you want, you can write further details about your dog’s behavior. ___________

Shyness/fearfulness

If you answer “I don’t know” to a question, the report of that part of the questionnaire will be empty.

Asterisk indicates mandatory field.

Data protection *
Hereby, I accept that my personal information, the information of my dog and all of the data collected with this questionnaire I have provided is transferred to Canine Genetics research group at the University of Helsinki and used in scientific research. Read the privacy policy of the Canine Genetics Group (only in Finnish).

Strangers
Answer how often your dog reacts as described below when meeting a stranger.

When meeting a stranger...
1. my dog withdraws *
   Never
   Rarely
   Sometimes
   Often
   Always or almost always
   I don't know

2. my dog barks *
   Never
   Rarely
   Sometimes
   Often
   Always or almost always
   I don’t know

3. my dog growls *
   Never
   Rarely
   Sometimes
   Often
   Always or almost always
   I don’n know

4. my dog keeps his/her tail low / between the legs. *
   Never

Rarely
Sometimes
Often
Always or almost always
I don’t know

5. my dog stays close to the owner (when not told to do so) *
Never
Rarely
Sometimes
Often
Always or almost always
I don’t know

6. my dog is not willing to make contact with stranger *
Never
Rarely
Sometimes
Often
Always or almost always
I don’t know

7. my dog greets strangers enthusiastically (e.g. jumps, licks face or hands) *
Never
Rarely
Sometimes
Often
Always or almost always
I don’t know

8. my dog is indifferent, but doesn't try to move away when a stranger tries to touch her/him *
Never
Rarely
Sometimes
Often
Always or almost always
I don’t know

9. my dog approaches a stranger with a low posture *
Never
Rarely
Sometimes

Often
Always or almost always
I don’t know

10. my dog approaches a stranger, but moves away if the stranger tries to touch her/him *
Never
Rarely
Sometimes
Often
Always or almost always
I don’t know

11. my dog is suspicious at the beginning, but greets the stranger later *
Never
Rarely
Sometimes
Often
Always or almost always
I don’t know

Unfamiliar dogs

Answer how often your dog reacts as described below when meeting an unfamiliar dog

When meeting an unfamiliar dog...

12. my dog barks *
Never
Rarely
Sometimes
Often
Always or almost always
I don’t know

13. my dog growls *
Never
Rarely
Sometimes
Often
Always or almost always
I don’t know

14. my dog keeps her/his tail low or between the legs *
Never

Rarely
Sometimes
Often
Always or almost always
I don’t know

15. my dog is not willing to make contact with the unfamiliar dog *
never
rarely
sometimes
often
always or almost always
I don’t know

16. my dog is enthusiastic *
Never
Rarely
Sometimes
Often
Always or almost always
I don’t know

17. my dog is indifferent, but doesn't try to move away from the other dog *
Never
Rarely
Sometimes
Often
Always or almost always
I don’t know

18. my dog approaches the unfamiliar dog with a low posture *
Never
Rarely
Sometimes
Often
Always or almost always
I don’t know

19. my dog moves away from the unfamiliar dog and wants to escape the situation *
Never
Rarely
Sometimes

Often
Always or almost always
I don't know

20. my dog tries to attack the unfamiliar dog *
Never
Rarely
Sometimes
Often
Always or almost always
I don't know

21. my dog is suspicious at the beginning, but greets the unfamiliar dog later *
Never
Rarely
Sometimes
Often
Always or almost always
I don't know

New situations / places

Answer how often your dog reacts as described below in a new situation or place.

In a new situation or place...
22. my dog keeps her/his tail low or between the legs *
Never
Rarely
Sometimes
Often
Always or almost always
I don't know

23. my dog pants *
Never
Rarely
Sometimes
Often
Always or almost always
I don't know

24. my dog wants out of the situation or place *

Never
Rarely
Sometimes
Often
Always or almost always
I don’t know

25. my dog trembles *
Never
Rarely
Sometimes
Often
Always or almost always
I don’t know

26. my dog stays close the owner (when not told to do so) *
Never
Rarely
Sometimes
Often
Always or almost always
I don’t know

27. my dog walks low to the ground *
Never
Rarely
Sometimes
Often
Always or almost always
I don’t know

28. my dog is restless and can't calm down *
Never
Rarely
Sometimes
Often
Always or almost always
I don’t know

29. my dog is curious and enthusiastic *
Never
Rarely

Sometimes
Often
Always or almost always
I don’t know

30. my dog is able to eat and sleep *
Never
Rarely
Sometimes
Often
Always or almost always
I don’t know

31. my dog is cautious at the beginning, but calms down later *
Never
Rarely
Sometimes
Often
Always or almost always
I don’t know

32. Do you feel that your dog shows shyness or fear when meeting a strange person? * (If answer is ”Yes”, Q32.1. is shown.)
Yes
No
I don't know

32.1. When did shyness toward strange people start? *
Before 3 months of age
At 3 months - 6 months of age
At 6 months - 1 year of age
At 1 year - 1.5 year of age
At 1.5 - 2 years of age
At 2 - 3 years of age
At 3 - 6 years of age
Older than 6 years old
The dog was shy before I got him/her

33. Has the shy behavior of dog towards strange people changed within the last six months? *
The dog's reaction has stayed the same
The dog has become fearful (wasn't before)
Fearfulness has increased

Fearfulness has decreased
The dog is no longer fearful at all

34. Do you feel that your dog shows shyness or fear when meeting an unfamiliar dog? * (If answer is ″Yes″, Q34.1. is shown.)
Yes
No
I don't know

34.1. When did shyness toward unfamiliar dogs start? *
Before 3 months of age
At 3 - 6 months of age
At 6 months - 1 year of age
At 1 - 1.5 year of age
At 1.5 - 2 years of age
At 2 - 3 years of age
At 3 - 6 years of age
Older than 6 years of age
The dog was shy before I got him/her

35. Has the shy behavior of dog towards strange dogs changed within the last six months? *
The dog's reaction has stayed the same
The dog has become fearful (wasn't before)
Fearfulness has increased
Fearfulness has decreased
The dog is no longer fearful at all

36. Do you feel that your dog shows fear or stress in a new situation or in a new environment? * (If answer is ″Yes″, Q36.1. is shown.)
Yes
No
I don't know

36.1. When did shyness toward new situations/environments start? *
Before 3 months of age
At 3 months - 6 months of age
At 6 months - 1 year of age
At 1 year - 1.5 year of age
At 1.5 - 2 years of age
At 2 - 3 years of age
At 3 - 6 years of age
Older than 6 years of age

The dog was shy before I got him/her

37. Has the shy behavior of dog towards new situation or places changed within the last six months? *

The dog's reaction has stayed the same

The dog has become fearful (wasn't before)

Fearfulness has increased

Fearfulness has decreased

The dog is no longer fearful at all

38. Does your dog have some other symptoms of shyness? Describe them. ____________________

39. Is your dog fearful of some other things? ________________________

40. If you want, you can write further details about your dog’s behavior. ____________________

Aggressiveness

The following questions deal with your dog's reaction towards strangers, the owner and family members, and other dogs. By stranger we mean a person your dog has never met before. By unfamiliar dog we mean a dog your dog has never met before.

If you answer "I don't know" to a question, the report of that part of the questionnaire will be empty.

Asterisk indicates mandatory field.

1. Data protection *
Hereby, I accept that my personal information, the information of my dog and all of the data collected with this questionnaire I have provided is transferred to Canine Genetics research group at the University of Helsinki and used in scientific research. Read the privacy policy of the Canine Genetics Group (only in Finnish).

Stranger
Please note how often the statement is true for your dog.

2. The dog barks when the doorbell rings or the door is knocked *
    Never
    Rarely
    Sometimes
    Often
    Always or almost always
    I don't know

3. The dog barks when strangers come in and at first does not want to greet *
    Never
    Rarely
    Sometimes
    Often
    Always or almost always
    I don't know

4. The dog barks when a stranger tries to touch or pet her/him in her/his home *
    Never
    Rarely
    Sometimes
    Often
    Always or almost always
    I don't know

5. The dog growls when a stranger tries to touch or pet her/him in her/his home *

Never
Rarely
Sometimes
Often
Always or almost always
I don't know

6. The dog tries to snap or bite when a stranger tries to touch or pet her/him in her/his home *

Never
Rarely
Sometimes
Often
Always or almost always
I don't know

7. The dog is happy and excited when strangers come to her/his home *

Never
Rarely
Sometimes
Often
Always or almost always
I don't know

8. When leashed outside the home, the dog barks when a stranger tries to touch or pet the dog. *

Never
Rarely
Sometimes
Often
Always or almost always
I don't know

9. When a stranger tries to pet the dog outside the home and the dog is on a leash, my dog growls at the stranger *

Never
Rarely
Sometimes
Often
Always or almost always
I don't know

10. When a stranger tries to pet the dog outside the home and the dog is on a leash, my dog tries to snap or bite the stranger *

Never
Rarely
Sometimes
Often
Always or almost always
I don't know

11. The dog barks or growls in the car at passersby *
Never
Rarely
Sometimes
Often
Always or almost always
I don't know

Owner/Family member

Please note how often the statement is true for your dog.

12. When the owner/family member handles (e.g. washes, grooms, cuts the nails) the dog, she/he growls *
Never
Rarely
Sometimes
Often
Always or almost always
I don't know

13. When the owner/family member handles (e.g. washes, grooms, cuts the nails) the dog, she/he tries to snap or bite *
Never
Rarely
Sometimes
Often
Always or almost always
I don't know

14. When the owner/family member takes a bone/food/toy from the dog, she/ he growls *
Never
Rarely
Sometimes
Often
Always or almost always
I don't know

15. When the owner/family member takes a bone/food/toy from the dog, she/he tries to snap or bite *
Never
Rarely
Sometimes
Often
Always or almost always
I don't know

Other dogs

Please note how often the statement is true for your dog.

16. When my dog meets an unfamiliar dog, my dog growls at it *
Never
Rarely
Sometimes
Often
Always or almost always
I don't know

17. When my dog meets an unfamiliar dog, my dog tries to attack it *
Never
Rarely
Sometimes
Often
Always or almost always
I don't know

18. When a familiar dog approaches my dog's food bowl/toy/treat, my dog growls *
Never
Rarely
Sometimes
Often
Always or almost always
I don't know

19. When a familiar dog approaches my dog's food bowl/toy/treat, my dog tries to attack the other dog *
Never
Rarely
Sometimes
Often
Always or almost always
I don't know

20. In your opinion, does your dog show aggressive behavior towards strange people? * (If answer is ”Yes”, Q20.1. is shown.)

Yes
No
I don't know

20.1. When did aggressiveness toward strange people start? *

Before 3 months of age
At 3 months - 6 months of age
At 6 months - 1 year of age
At 1 year - 1.5 year of age
At 1.5 - 2 years of age
At 2 - 3 years of age
At 3 - 6 years of age
Older than 6 years of age
The dog showed already aggressive behavior when I got him/her

21. Has your dog's reaction to strangers changed within the last six months? *

The dog's reaction has stayed the same
The dog has become aggressive (wasn't before)
Aggressiveness has increased
Aggressiveness has decreased
The dog is no longer aggressive at all

22. In your opinion, does your dog show aggressive behavior toward owner/family member? * (If answer is ”Yes”, Q22.1. is shown.)

Yes
No
I don't know

22.1. When did aggressiveness toward owner/family member start? *

Before 3 months of age
At 3 months - 6 months of age
At 6 months - 1 year of age
At 1 year - 1.5 year of age
At 1.5 - 2 years of age
At 2 - 3 years of age
At 3 - 6 years of age
Older than 6 years of age
The dog showed already aggressive behavior when I got him/her

23. Has your dog's reaction to owner/family member changed within the last six months? *

The dog's reaction has stayed the same
The dog has become aggressive (wasn't before)
Aggressiveness has increased
Aggressiveness has decreased
The dog is no longer aggressive at all

24. In your opinion, does your dog show aggressive behavior toward other dogs? * (If answer is "Yes", Q24.1. is shown.)
Yes
No
I don't know

24.1. When did aggressiveness toward other dogs start? *
Before 3 months of age
At 3 months - 6 months of age
At 6 months - 1 year of age
At 1 year - 1.5 year of age
At 1.5 - 2 years of age
At 2 - 3 years of age
At 3 - 6 years of age
Older than 6 years of age
The dog showed already aggressive behavior when I got him/her

25. Has your dog's reaction to other dogs changed within the last six months? *
The dog's reaction has stayed the same
The dog has become aggressive (wasn't before)
Aggressiveness has increased
Aggressiveness has decreased
The dog is no longer aggressive at all

26. Does your dog have some other symptoms of shyness? Describe them.
____

27. Does your dog show aggressiveness in some other situations? ____________

28. If you want, you can write further details about your dog’s behavior. _____________

Surface phobia

If you answer "I don't know" to a question, the report of that part of the questionnaire will be empty.

Asterisk indicates mandatory field

1. Data protection *

Hereby, I accept that my personal information, the information of my dog and all of the data collected with this questionnaire I have provided is transferred to Canine Genetics research group at the University of Helsinki and used in scientific research. Read the privacy policy of the Canine Genetics Group (only in Finnish).

Surfaces and high places - Does your dog have difficulties to walk...

2. …on a metal grid? *
   Yes, always or often
   Sometimes, depends on the place
   Never
   I don't know
   My dog has not encountered this situation

3. …on shiny floors (like in shopping malls, at veterinary clinics)? *
   Yes, always or often
   Sometimes, depends on the place
   Never
   I don't know
   My dog has not encountered this situation
4. …on stairs where you can see 'between' the steps (open riser stairs)? *
   Yes, always or often
   Sometimes, depends on the place
   Never
   I don't know
   My dog has not encountered this situation

5. …on stairs where you cannot see 'between' the steps (closed risers)? *
   Yes, always or often
   Sometimes, depends on the place
   Never
   I don't know
   My dog has not encountered this situation

6. …next to glass railings, e.g. on the second floor in a shopping mall? *
   Yes, always or often

Sometimes, depends on the place
Never
I don't know
My dog has not encountered this situation

7. …on metal stairs where you can see 'through' the steps? *
Yes, always or often
Sometimes, depends on the place
Never
I don't know
My dog has not encountered this situation

8. …on narrow bridges? *
Yes, always or often
Sometimes, depends on the place
Never
I don't know
My dog has not encountered this situation

9. …from one surface to another (e.g. from outside to inside in a new place)? *
Yes, always or often
Sometimes, depends on the place
Never
I don't know
My dog has not encountered this situation

10. …on a slippery floor? *
Yes, always or often
Sometimes, depends on the place
Never
I don't know
My dog has not encountered this situation

11. …on agility contact obstacles (A-frame, dogwalk, teeter-totter)? *
Yes, always or often
Sometimes, depends on the place
Never
I don't know
My dog has not encountered this situation

12. Do you feel your dog has difficulties to walk on different surfaces? * (If answer is "Yes", Q12.1. is shown.)
Yes

No
I don't know

12.1. When did your dog first start to react to surfaces and high places? *
Before 3 months of age
At 3 months - 6 months of age
At 6 months - 1 year of age
At 1 year - 1.5 year of age
At 1.5 - 2 years of age
At 2 - 3 years of age
At 3 - 6 years of age
Older than 6 years of age
The dog had fear of surfaces before I got him/her

13. Has your dog's reaction to **surfaces/heights** changed **within the last six months**? *
The dog's reaction has stayed the same
The dog has become fearful of surfaces/heights (wasn't before)
Surface phobia has increased
Surface phobia has decreased
The dog is no longer fearful of surfaces/heights at all

14. How does your dog react to heights and different surfaces? Describe your dog’s behavior. _______

15. Is your dog fearful on some other surfaces? __________

16. If you want, you can write further details about your dog’s behavior. ______

Separation anxiety

The following questions deal with dog's behaviour when s/he is alone without human present. If you answer “I don’t know” to a question, the report of that part of the questionnaire will be empty.

Asterisk indicates mandatory field.

1. Data protection *
Hereby, I accept that my personal information, the information of my dog and all of the data collected with this questionnaire I have provided is transferred to Canine Genetics research group at the University of Helsinki and used in scientific research. Read the privacy policy of the Canine Genetics Group (only in Finnish).

Please answer how often the following statements are true for your dog.
2. The dog vocalizes (e.g. whines) when the owner is leaving *
    Never
    Rarely
    Sometimes
    Often
    Very often
    I don't know

3. The dog is restless when the owner is leaving *
    Never
    Rarely
    Sometimes
    Often
    Very often
    I don't know

4. The dog pants when the owner is leaving *
    Never
    Rarely
    Sometimes
    Often
    Very often
    I don't know

5. The dog salivates when the owner is leaving *
    Never
    Rarely
    Sometimes

Often
Very often
I don't know

6. The dog destroys / chews things when home alone *
Never
Rarely
Sometimes
Often
Very often
I don't know

7. The dog urinates or defecates when home alone *
Never
Rarely
Sometimes
Often
Very often
I don't know

8. The dog vocalizes (e.g. howls, barks) when home alone *
Never
Rarely
Sometimes
Often
Very often
I don't know

9. The dog salivates when home alone *
Never
Rarely
Sometimes
Often
Very often
I don't know

10. The dog pants when home alone *
Never
Rarely
Sometimes
Often
Very often

I don't know

11. Do you feel that your dog exhibits separation anxiety when left alone or left with other dogs? * (If answer is ”Yes”, Q11.1 is shown.)

Yes

No

I don't know

11.1. When did separation anxiety begin? *

Before 3 months of age

At 3 months - 6 months of age

At 6 months - 1 year of age

At 1 year - 1.5 year of age

At 1.5 - 2 years of age

At 2 - 3 years of age

At 3 - 6 years of age

Older than 6 years of age

The dog had separation anxiety before I got her/him

12. How do you know about your dog's reactions when home alone? *

I have stayed outside of my apartment for a while to listen/observe

I have videotaped the dog

I monitor my dog's behavior real-time via some software

My neighbours have told me

I read the dog's mood when I come home

The dog has destroyed something when alone

Something else (If ticked, the Q12.1 is shown: Q12.1. Please specify:____)

13. Has your dog's reaction to alone time changed within the last six months? *

The dog is no longer fearful of being alone at all

Separation anxiety has decreased

Separation anxiety has increased

The dog has become fearful of being alone (wasn't before)

The dog's reaction has stayed the same

14. Does your dog have some other symptoms of separation anxiety? Describe them.

____

15. If you want, you can write further details about your dog’s behavior. ____________________________________

Impulsivity/inattention

If you answer "I don't know" to a question, the report of that part of the questionnaire will be empty.

Asterisk indicates mandatory field.

Data protection *
Hereby, I accept that my personal information, the information of my dog and all of the data collected with this questionnaire I have provided is transferred to Canine Genetics research group at the University of Helsinki and used in scientific research. Read the privacy policy of the Canine Genetics Group (only in Finnish).

Please note how often the statement is true for your dog.
1. My dog has a difficult time learning, because she/he is careless or other things can easily attract her/his attention. *
Never
Sometimes
Often
Very often

2. It's easy to attract my dog's attention, but she/he loses interest soon. *
Never
Sometimes
Often
Very often

3. It's difficult for my dog to concentrate on a task or play. *
Never
Sometimes
Often
Very often

4. My dog leaves from her/his place when she/he should stay. *
Never
Sometimes
Often
Very often

5. My dog cannot be quiet or easily calmed. *
Never
Sometimes
Often
Very often

6. My dog fidgets all the time. *

Never
Sometimes
Often
Very often

7. It seems that my dog doesn't listen even if she/he knows that someone is speaking to her/him. *

Never
Sometimes
Often
Very often

8. My dog is excessive, difficult to control, and if she/he lunges it is hard to hold back. *

Never
Sometimes
Often
Very often

9. My dog would always play and run. *

Never
Sometimes
Often
Very often

10. My dog solves simple tasks easily, but she/he often has difficulties with complicated tasks, even if she/he knows them and has practiced them often. *

Never
Sometimes
Often
Very often

11. My dog is likely to react hastily and that's why she/he is failing tasks. *

Never
Sometimes
Often
Very often

12. My dog's attention can be easily distracted. *

Never
Sometimes
Often
Very often

13. My dog cannot wait as she/he has no self-control. *

Never
Sometimes
Often
Very often

14. If you want, you can write further details about your dog's behavior. Include the number of the statement the comment is referring to. ___________________

Please note how often the statement is true for your dog.

15. My dog shows extreme physical signs when excited (e.g. drooling, panting, raising hackles, urination, licking lips, widening of eyes) *

Strongly disagree
Generally disagree
Partly agree, partly disagree
Generally agree
Strongly agree

16. When my dog gets very excited it can lead to fixed repetitive behavior (i.e., an action that is repeated in the same way over and over again), such as tail chasing or spinning around in circles *

Strongly disagree
Generally disagree
Partly agree, partly disagree
Generally agree
Strongly agree

17. I would consider my dog to be very impulsive (i.e., has sudden, strong urges to act; acts without forethought; acts without considering effects of actions) *

Strongly disagree
Generally disagree
Partly agree, partly disagree
Generally agree
Strongly agree

18. My dog doesn't like to be approached or hugged *

Strongly disagree
Generally disagree
Partly agree, partly disagree
Generally agree
Strongly agree

19. My dog becomes aggressive (e.g. growls, snarls, snaps or bites) when excited *

Strongly disagree
Generally disagree
Partly agree, partly disagree
Generally agree
Strongly agree

20. My dog appears to be 'sorry' after she/he has done something wrong *
Strongly disagree
Generally disagree
Partly agree, partly disagree
Generally agree
Strongly agree

21. My dog does not think before she/he acts (e.g. would steal food without first looking to see if someone is watching) *
Strongly disagree
Generally disagree
Partly agree, partly disagree
Generally agree
Strongly agree

22. My dog can be very persistent (e.g. will continue to do something even if she/he knows she/he will get punished or told off) *
Strongly disagree
Generally disagree
Partly agree, partly disagree
Generally agree
Strongly agree

23. My dog may become aggressive (e.g. growls, snarls, snaps or bites) if frustrated with something *
Strongly disagree
Generally disagree
Partly agree, partly disagree
Generally agree
Strongly agree

24. My dog is easy to train *
Strongly disagree
Generally disagree
Partly agree, partly disagree
Generally agree
Strongly agree

25. My dog is not keen to go into new situations *
Strongly disagree
Generally disagree
Partly agree, partly disagree
Generally agree
Strongly agree

26. My dog takes a long time to lose interest in new things *
Strongly disagree
Generally disagree
Partly agree, partly disagree
Generally agree
Strongly agree

27. My dog calms down very quickly after being excited *
Strongly disagree
Generally disagree
Partly agree, partly disagree
Generally agree
Strongly agree

28. My dog appears to have a lot of control over how she/he responds *
Strongly disagree
Generally disagree
Partly agree, partly disagree
Generally agree
Strongly agree

29. My dog is very interested in new things and new places *
Strongly disagree
Generally disagree
Partly agree, partly disagree
Generally agree
Strongly agree

30. My dog reacts very quickly *
Strongly disagree
Generally disagree
Partly agree, partly disagree
Generally agree
Strongly agree

31. My dog is not very patient (e.g. gets agitated waiting for her/his food, or waiting to go out for a walk) *
Strongly disagree
Generally disagree
Partly agree, partly disagree
Generally agree
Strongly agree

32. My dog seems to get excited for no reason *
Strongly disagree
Generally disagree
Partly agree, partly disagree
Generally agree
Strongly agree

33. If you want, you can write further details about your dog's behavior. Include the number of the statement the comment is referring to.

34. Do you feel that your dog is hyperactive/impulsive? * (If answer is "Yes", Q34.1. is shown.)
Yes
No
I don't know

34.1. When did hyperactive or impulsive behavior begin? *
Before 3 months of age
At 3 months - 6 months of age
At 6 months - 1 year of age
At 1 year - 1.5 year of age
At 1.5 - 2 years of age
At 2 - 3 years of age
At 3 - 6 years of age
Older than 6 years of age
The dog was hyperactivity/impulsive before I got him/her

35. My dog's hyperactivity/impulsiveness disturbs daily routines and/or training of the dog *
Never
Rarely
Sometimes
Often
Always or almost always
I don't know

36. Has your dog's activity/impulsivity changed within the last six months? *

The dog's activity/impulsivity has stayed the same
Activity/impulsivity has increased
Activity/impulsivity has decreased

37. Has your dog's attention span changed within the last six months? *
The dog's attention span has stayed the same
Attention span has improved
Attention span has weakened

Cognition (perception, learning and memory)

The following questions deal with your dog's cognition: perception, recognition, learning, and memory. At an old age, changes in these traits can be an indication of canine cognitive dysfunction. If more than one option matches your dog, please select the last matching option.

Asterisk indicates mandatory field.

Data protection *
Hereby, I accept that my personal information, the information of my dog and all of the data collected with this questionnaire I have provided is transferred to Canine Genetics research group at the University of Helsinki and used in scientific research. Read the privacy policy of the Canine Genetics Group (only in Finnish).

1. Appetite *
    - Normal
    - Decreased (if ticked, Q.1.1. is shown)
    - Increased with diarrhea (if ticked, Q.1.1. is shown)
    - Increased without diarrhea (if ticked, Q.1.1. is shown)

    1.1. How often there are changes in the appetite of your dog?
    - Around once per month
    - Around once per week
    - Around once per day
    - Multiple times per day

2. Drinking *
    - Normal
    - Polydipsia (excessive drinking) (if ticked, Q.2.1. is shown)

    2.1. How often dog drinks more than normally?
    - Around once per month
    - Around once per week
    - Around once per day
    - Multiple times per day

3. Urinating indoors not caused by incontinence (select last matching option) *
    - Does not urinate indoors
    - Urinates indoors (if ticked, Q.3.1. is shown)
    - Urinates and defecates in the house (if ticked, Q.3.2. is shown)

    3.1. How often does dog urinate indoors?

Around once per month
Around once per week
Around once per day
Multiple times per day

3.2. How often does dog urinate/defecate indoors?
Around once per month
Around once per week
Around once per day
Multiple times per day

4. Day/night rhythm (select last matching option) *
Normal
Sleeping increased (if ticked, Q.4.1. is shown)
Sleeps at day, restless at night (if ticked, Q.4.1. is shown)

4.1. How often there are disturbances in your dog's day rhythm?
Around once per month
Around once per week
Around once per day
Multiple times per day

5. Aimless behavior (select last matching option) *
No aimless behavior
Stares blankly at walls/floor (if ticked, Q.5.1. is shown)
Paces or wanders with no direction or purpose (if ticked, Q.5.1. is shown)
Walks in circles (if ticked, Q.5.1. is shown)

5.1. How often does your dog show aimless behavior?
Around once per month
Around once per week
Around once per day
Multiple times per day

6. Interaction with people and environment *
Normal
Decreased (if ticked, Q.6.1. is shown)
No contact with the environment/owner (if ticked, Q.6.1. is shown)

6.1. How often there are disturbances in your dog's interaction?
Around once per month
Around once per week

Around once per day
Multiple times per day

7. Loss of perception (select last matching option) *
No loss of perception
Collides into furniture (if ticked, Q.7.1. is shown)
Tries to pass through too narrow spaces (if ticked, Q.7.1. is shown)
Tries to pass through the wrong side of the door (if ticked, Q.7.1. is shown)

7.1. How often there are disturbances in the perception?
Around once per month
Around once per week
Around once per day
Multiple times per day

8. Disorientation (select last matching option) *
No disorientation
On new routes (if ticked, Q.8.1. is shown)
On daily familiar routes (if ticked, Q.8.1. is shown)
At home (if ticked, Q.8.1. is shown)

8.1. How often does your dog show signs of disorientation?
Around once per month
Around once per week
Around once per day
Multiple times per day

9. Memory (select last matching option) *
Normal
No recognition of acquaintances (if ticked, Q.9.1. is shown)
No recognition of the owner after a holiday (if ticked, Q.1.9.1. is shown)
No recognition of the owner at a daily basis (if ticked, Q.9.1. is shown)

9.1. How often there are issues with your dogs memory?
Around once per month
Around once per week
Around once per day
Multiple times per day

10. Personality changes (select last matching option) *
No changes in personality
Aggressive towards other pets/children (if ticked, Q.10.1. is shown)

Aggressive towards the owner (if ticked, Q.10.1. is shown)

10.1. How often there are changes in the personality?

Around once per month

Around once per week

Around once per day

Multiple times per day

11. Difficulty finding dropped food (select last matching option) *

No difficulties in finding food

Finds food after a somewhat long search (if ticked, Q.11.1. is shown)

Searches for food but does not find it (if ticked, Q.11.1. is shown)

Is not interested in searching for food (if ticked, Q.11.1. is shown)

11.1. How often does your dog show difficulties in finding dropped food?

Around once per month

Around once per week

Around once per day

Multiple times per day

Has your dog displayed following behaviors when they have been under 5 years old?

12. Fear towards strangers

No

Mild

Moderate

Severe

13. Fear towards strange dogs

No

Mild

Moderate

Severe

14. Fear towards novel situations

No

Mild

Moderate

Severe

15. Aggressiveness towards strangers

No

Mild

Moderate
Severe

16. Aggressiveness towards family members
No
Mild
Moderate
Severe

17. Aggressiveness towards other dogs
No
Mild
Moderate
Severe

18. Noise phobia towards fireworks
No
Mild
Moderate
Severe

19. Noise phobia towards thunder
No
Mild
Moderate
Severe

20. Noise phobia towards other sounds
No
Mild
Moderate
Severe

21. Fear of surfaces/heights
No
Mild
Moderate
Severe

22. Separation anxiety
No
Mild

Moderate
Severe

23. Hyperactivity/impulsivity
No
Mild
Moderate
Severe

24. Inattention
No
Mild
Moderate
Severe

25. Stereotypic tailchasing
No
Mild
Moderate
Severe

26. Stereotypic chasing of reflections/shadows
No
Mild
Moderate
Severe

27. Stereotypic pacing/staring
No
Mild
Moderate
Severe

28. Other stereotypical behavior
No
Mild
Moderate
Severe

29. Have you noticed some other changes in your dog's cognitive capabilities? Describe them. ________________

30. If you want, you can write further details about your dog’s behavior. _______________

Stereotypic behavior

If you answer "I don't know" to a question, the report of that part of the questionnaire will be empty.

Asterisk indicates mandatory field.

Data protection *
Hereby, I accept that my personal information, the information of my dog and all of the data collected with this questionnaire I have provided is transferred to Canine Genetics research group at the University of Helsinki and used in scientific research. Read the privacy policy of the Canine Genetics Group (only in Finnish).

1. Tail-chasing/spinning - The dog chases his/her tail or spins repeatedly. The dog may stop and stare at his/her tail/rear. Does your dog chase his/her tail or spin repeatedly? * (If answer is "More often (at least once a year)", Q1.1-Q1.9. are shown.)
    I've never noticed this behavior
    A few times during the dog's lifetime
    More often (at least once a year)

    1.1. Please specify, how often your dog chases his/her tail or spins. *
        Every once in a while (monthly-yearly)
        Quite often (weekly-monthly)
        Repeatedly (every other day-weekly)
        Daily
        Several times per day

    1.2. Describe a typical tail-chasing/spinning episode ___________________

    1.3. How long on average does your dog spend tail-chasing/spinning during one day? *
        0 - 30 min
        30 min - 1 hour
        1 - 2 hours
        2 - 5 hours
        5 hours or more

    1.4. On average, how much time does the dog's one tail-chasing/spinning episode last? *
        0 - 5 seconds
        5 - 30 seconds
        30 seconds - 1 min
        1 min - 5 min
        5 min - 10 min
        10 min - 15 min

15 min - 1 hour
More than 1 hour

1.5. Does tail-chasing/spinning negatively affect the dog's daily activities or other functions? * (if answer is “Yes”, Q1.5.1. is shown.)

Yes
No
I don't know

1.5.1. Please specify, how: ___________

1.6. Have you told (ordered/commanded) your dog to stop chasing tail/spinning? * (If answer is “Yes”, Q1.6.1. is shown.)

Yes
No
I don't remember

1.6.1. Does your dog stop chasing his/her tail or spinning when requested? *

Yes, most of the time
Yes, sometimes
Yes, but soon starts again e.g. in another room
No

1.7. During tail-chasing/spinning, does the dog react to his/her name or other commands? *

Yes, like normally
Yes, but worse than normally
Not usually
Not at all

1.8. Does tail-chasing/spinning occur during a specific situation? You can choose multiple options. *

The behavior does not occur during any specific situation
Excitement/arousal
Going for/coming back from a walk
Playing, play-fighting
Stress, frustration
The dog is bored, has too much energy
Arrival of the owner or a guest
The behavior occurs during a specific time of the day
Something else (if ticked, Q1.8.1. is shown)

1.8.1. Please specify: _________

1.9. When did tail-chasing/spinning begin? *
Before 3 months of age
At 3 months - 6 months of age
At 6 months - 1 year of age
At 1 year - 1.5 year of age
At 1.5 - 2 years of age
At 2 - 3 years of age
At 3 - 6 years of age
Older than 6 years of age
My dog had stereotypic behavior before I got him/her

2. Reflections and shadows - The dog stares/chases reflections or shadows. Does your dog pursue, snatch or bite at reflections or shadows? * (If answer is "More often (at least once a year)", Q2.1-Q2.9. are shown.)
I've never noticed this behavior
A few times during the dog's lifetime
More often (at least once a year)

2.1. Please specify, how often your dog pursues reflections or shadows. *
Every once in a while (monthly-yearly)
Quite often (weekly-monthly)
Repeatedly (every other day-weekly)
Daily
Several times per day

2.2. Describe a typical episode of your dog pursuing reflections/shadows ____________

2.3. How long on average does your dog spend pursuing reflections/shadows during one day? *
0 - 30 min
30 min - 1 hour
1 - 2 hours
2 - 5 hours
5 hours or more

2.4. On average, how long does one pursuing episode generally last? *
0 - 5 seconds
5 - 30 seconds
30 seconds - 1 min
1 min - 5 min
5 min - 15 min
15 min - 1 hour
1 - 2 hours
2 hours or more

2.5. Does pursuing reflections/shadows negatively affect the dog's daily activities or other functions? * (If answer is "Yes", Q2.5.1. is shown.)

Yes

No

I don't know

2.5.1. Please specify: __________

2.6. Have you told (ordered/commanded) your dog to stop pursuing reflections/shadows? * (If answer is "Yes", Q2.6.1. is shown.)

Yes

No

I don't remember

2.6.1. Does your dog stop pursuing reflections/shadows when requested? *

Yes, most of the time

Yes, sometimes

Yes, but soon starts again e.g. in another room

No

2.7. During pursuing reflections/shadows, does your dog react to his/her name or other commands? *

Yes, like normally

Yes, but worse than normally

Not usually

Not at all

2.8. Does pursuing reflections/shadows occur during a specific situation? You can choose multiple options. *

The behavior does not occur during any specific situation

Excitement/arousal

Going for/coming back from a walk

Playing, play-fighting

Stress, frustration

The dog is bored, has too much energy

Arrival of the owner or a guest

Darkness

Sunshine

Something else (if ticked, Q2.8.1. is shown)

2.8.1. Please specify: _________

2.9. When did pursuing reflections/shadows begin? *

Before 3 months of age

At 3 months - 6 months of age
At 6 months - 1 year of age
At 1 year - 1.5 year of age
At 1.5 - 2 years of age
At 2 - 3 years of age
At 3 - 6 years of age
Older than 6 years of age
My dog had stereotypic behavior before I got him/her

3. Invisible things - The dog looks like it is trying to catch invisible things. Does your dog pursue invisible things? * (If answer is ”More often (at least once a year)”, Q3.1-Q3.9. are shown.)
I've never noticed this behavior
A few times during the dog's lifetime
More often (at least once a year)

3.1. Please, specify how often your dog chases invisible things. *
Every once in a while (monthly - yearly)
Quite often (weekly - monthly)
Repeatedly (every other day - weekly)
Daily
Several times per day

3.2. Describe a typical chasing of invisible things episode: _______________

3.3. How long on average does your dog spend chasing invisible things during one day? *
0 - 30 min
30 min - 1 hour
1 - 2 hours
2 - 5 hours
5 hours or more

3.4. On average, how much time does your dog's one chasing of invisible things episode last? *
0 - 5 sec
5 - 30 sec
30 sec - 1 min
1 - 5 min
5 - 10 min
10 - 15 min
15 min - 1 hour
more than 1 hour

3.5. Does chasing of invisible things negatively affect your dog's daily activities or other functions? * (If answer is "Yes", Q3.5.1. is shown.)

Yes

No

I don't know

3.5.1. Please, specify: _________

3.6. Have you told (ordered/commanded) your dog to stop chasing invisible things? * (If answer is "Yes", Q3.6.1. is shown.)

Yes

No

I don't remember

3.6.1. Does your dog stop chasing of invisible things when requested? *

Yes, most of the time

Yes, sometimes

Yes, but soon starts again e.g. in another room

No

3.7. During chasing of invisible things, does your dog react to his/her name or other commands? *

Yes, like normally

Yes, but worse than normally

Not usually

Not at all

3.8. Does chasing invisible things occur during a specific situation? You can select multiple options. *

The behavior does not occur during any specific situation

Going for/coming back from a walk

Excitement/arousal

Playing, play-fighting

Stress, frustration

The dog is bored, has too much energy

Arrival of the owner or a guest

The behavior occurs during a specific time of the day

Something else (if ticked, Q3.8.1. is shown)

3.8.1. Please specify: _______

3.9. When did chasing of invisible things begin? *

Before 3 months of age

At 3 months - 6 months of age

At 6 months - 1 year of age
At 1 year - 1.5 year of age
At 1.5 - 2 years of age
At 2 - 3 years of age
At 3 - 6 years of age
Older than 6 years of age
My dog had stereotypic behavior before I got him/her

4. Licking surfaces - The dog licks different sufaces e.g. floors or walls for a long time although there is no smell of food. Does your dog lick surfaces? * (If answer is ″More often (at least once a year)″, Q4.1-Q4.9. are shown.)
I've never noticed this behavior
a few times during the dog's lifetime
more often (at least once a year)

4.1. Please, specify how often dog licks surfaces in a year. *
every once in a while (monthly-yearly)
quite often (weekly-monthly)
repeatedly (every other day-weekly)
daily
several times per day

4.2. Describe a typical surface licking episode: ___________

4.3. How long on average does your dog spend licking surfaces during one day? *
0 - 30 min
30 min - 1 hour
1 - 2 hours
2 - 5 hours
5 hours or more

4.4. On average, how much time does your dog spend licking without stopping? *
0 - 5 seconds
5 - 30 seconds
30 seconds - 1 min
1 min - 5 min
5 min - 15 min
15 min - 1 hours
1 - 2 hours
2 hours or more

4.5. Does licking surfaces negatively affect your dog's daily activities or other functions? * (If answer is "Yes", Q4.5.1.)
Yes

No
I don't know

4.5.1. Please specify: ____________

4.6. Have you told (ordered/commanded) your dog to stop licking surfaces? * (If answer is "Yes", Q4.6.1. is shown.)
Yes
No
I don't remember

4.6.1. Does your dog stop licking of surfaces when requested? *
Yes, most of the time
Yes, sometimes
Yes, but soon starts again e.g. in another room
No

4.7. During licking of surfaces, does your dog react to his/her name or other commands? *
Yes, like normally
Yes, but worse than normally
Not usually
Not at all

4.8. Does licking surfaces occur during a specific situation? You can choose multiple options. *
The behavior does not occur during any specific situation
During meals
Excitement/arousal
Going for/coming back from a walk
Playing, play-fighting
Stress, frustration
The dog is bored, has too much energy
Arrival of the owner or a guest
Before going to sleep or rest
The behavior occurs during a specific time of the day
Acid reflux, nausea or other discomfort/pain
Something else (if ticked, Q4.8.1. is shown)

4.8.1. Please specify: ____________

4.9. When did licking surfaces begin? *
Before 3 months of age
At 3 months - 6 months of age

At 6 months - 1 year of age
At 1 year - 1.5 year of age
At 1.5 - 2 years of age
At 2 - 3 years of age
At 3 - 6 years of age
Older than 6 years of age
My dog had stereotypical behavior before I got him/her

5. Pacing - Pacing means schematic (e.g. circle, figure 8's) or "aimless" running/walking. The dog can e.g. pace back and forth next to a wall. Does your dog pace? * (If answer is ”More often (at least once a year)”, Q5.1-Q5.9. are shown.)
I've never noticed this behavior
A few times during the dog's lifetime
More often (at least once a year)

5.1. Please specify, how often your dog paces. *
every once in a while (monthly-yearly)
quite often (weekly-monthly)
repeatedly (every other day-weekly)
daily
several times per day

5.2. Describe a typical pacing episode: _______________

5.3. How long on average does your dog pace during one day? *
0 - 30 min
30 min - 1 hour
1 - 2 hours
2 - 3 hours
3 - 5 hours
5 hours or more

5.4. On average, how long does your dog's one pacing episode last? *
0 - 5 seconds
5 - 30 seconds
30 seconds - 1 min
1 min - 5 min
5 min - 15 min
15 min - 1 hour
1 - 2 hour
2 hours or more

5.5. Does pacing negatively affect your dog's daily activities or other functions? * (If answer is "Yes", Q5.5.1. is shown.)

No

Yes

I don't know

5.5.1. Please specify, how: _____________

5.6. Have you told (ordered/commanded) your dog to stop pacing? * (If answer is "Yes", Q5.6.1. is shown.)

Yes

No

I don't remember

5.6.1.Does your dog stop pacing when requested? *

Yes, most of the time

Yes, sometimes

Yes, but soon starts again e.g. in another room

No

5.7. During pacing, does your dog react to his/her name or other commands? *

Yes, like normally

Yes, but worse than normally

Not usually

Not at all

5.8. Does pacing occur during a specific situation? You can choose multiple options. *

The behavior does not occur during any specific situation

During meals

Excitement/arousal

Going for/coming back from a walk

Playing, play-fighting

Stress, frustration

The dog is bored, has too much energy

Arrival of the owner or a guest

The behavior occurs during a specific time during the day

Something else (if ticked, Q5.8.1. is shown)

5.8.1. Please specify: __________

5.9. When did pacing begin? *

Before 3 months of age

At 3 months - 6 months of age

At 6 months - 1 year of age
At 1 year - 1.5 year of age
At 1.5 - 2 years of age
At 2 - 3 years of age
At 3 - 6 years of age
Older than 6 years of age
My dog had stereotypic behavior before I got him/her

6. Staring - Dog stares at one spot for a very long time, even if nothing is there. The dog may stare at e.g. wall or ceiling. Does your dog stare at a specific spot for long periods? * (If answer is ”More often (at least once a year)”, Q6.1-Q6.9. are shown.)
I've never noticed this behavior
A few times during the dog's lifetime
More often (at least once a year)

6.1. Please specify how often staring happens. *
every once in a while (monthly-yearly)
quite often (weekly-monthly)
repeatedly (every other day-weekly)
daily
several times per day

6.2. Describe a typical staring episode _______________

6.3. How long on average does your dog spend staring during one day? *
0 - 30 min
30 min - 1 hours
1 - 2 hours
2 - 3 hours
3 - 5 hours
5 hours or more

6.4. On average, how long does your dog's one staring episode last? *
0 - 5 seconds
5 - 30 seconds
30 seconds - 1 min
1 min - 5 min
5 min - 10 min
10 min – 15 min
15 min – 30 min
30 min – 1 hour
1 hour – 2 hours

2 hours or more

6.5. Does staring negatively affect your dog's daily activities or other functions? * (If answer is ″Yes″, Q6.5.1. is shown.)

Yes

No

I don't know

6.5.1. Please specify: _____________

6.6. Have you told (ordered/commanded) your dog to stop staring? * (If answer is ″Yes″, Q6.6.1. is shown.)

Yes

No

I don't remember

6.6.1. Does your dog stop staring when requested? *

Yes, most of the time

Yes, sometimes

Yes, but soon starts again e.g. in another room

No

6.7. During staring, does your dog react to his/her name or other commands? *

Yes, like normally

Yes, but worse than normally

Not usually

Not at all

6.8. Does staring occur during a specific situation? You can choose multiple options. *

The behavior does not occur during any specific situation

Playing, play-fighting

Has seen an animal and continues staring after the animal has left

Excitement/arousal

Stress, frustration

The dog is bored, has too much energy

Arrival of the owner or a guest

The behavior occurs during a specific time of the day

Something else (if ticked, Q6.8.1. is shown)

6.8.1. Please specify: _____________________

6.9. When did staring begin? *

Before 3 months of age

At 3 months - 6 months of age
At 6 months - 1 year of age
At 1 year - 1.5 year of age
At 1.5 - 2 years of age
At 2 - 3 years of age
At 3 - 6 years of age
Older than 6 years of age
My dog had stereotypic behavior before I got him/her

7. Excessive drinking - Dog drinks too much water or plays with the water bowl constantly. In your opinion, does your dog drink too much or spend too much time near the water bowl? * (If answer is "More often (at least once a year)", Q7.1-Q7.9. are shown.)
I've never noticed this behavior
A few times during the dog's lifetime
More often (at least once a year)

7.1. Please specify how often your dog drinks too much. *
Every once in a while (monthly-yearly)
Quite often (weekly-monthly)
Repeatedly (every other day-weekly)
Daily
Several times per day

7.2. Describe a typical excessive drinking episode _______________

7.3. How long on average does your dog spend drinking excessively during one day? *
0 - 30 min
30 min - 1 hour
1 - 2 hours
2 - 5 hours
5 hours or more

7.4. On average, how long does your dog's one drinking episode last? *
0 - 5 sec
5 - 30 sec
30 sec - 1 min
1 min - 5 min
5 min - 10 min
10 min - 15 min
15 min - 1 hour
more than 1 hour

7.5. Does excessive drinking negatively affect your dog's daily activities or other functions? * (If answer is ”Yes”, Q7.5.1. is shown.)

Yes

No

I don't know

7.5.1. Please specify, how: ______________

7.6. Have you told (ordered/commanded) your dog to stop drinking water? * (If answer is ”Yes”, Q7.6.1. is shown.)

Yes

No

I don't remember

7.6.1. Does your dog stop drinking when requested? *

Yes, most of the time

Yes, sometimes

Yes, but soon starts again e.g. in another room

No

7.7. During drinking, does your dog react to his/her name or other commands? *

Yes, like normally

Yes, but worse than normally

Not usually

Not at all

7.8. Does excessive drinking occur during a specific situation? You can choose multiple options. *

The behavior does not occur during any specific situation

During meals

Going for/coming back from a walk

Stress, frustration

Excitement/arousal

The dog is bored, has too much energy

Playing, play-fighting

Before going to sleep or rest

Behavior occurs during a specific time of the day

Something else (if ticked, Q7.8.1. is shown)

7.8.1. Please specify: ________________

7.9. When did excessive drinking begin? *

Before 3 months of age

At 3 months - 6 months of age
At 6 months - 1 year of age
At 1 year - 1.5 year of age
At 1.5 - 2 years of age
At 2 - 3 years of age
At 3 - 6 years of age
Older than 6 years of age
My dog had stereotypic behavior before I got him/her

8. Chewing or licking of body parts - Dog excessively chews or licks some parts of her/his body. Does your dog chew or lick herself/himself? * (If answer is ″More often (at least once a year)″, Q8.1-Q8.10. are shown.)
I've never noticed this behavior
A few times during the dog's lifetime
More often (at least once a year)

8.1. Please specify, how often your dog chews or licks himself/herself excessively. *
Every once in a while (monthly-yearly)
Quite often (weekly-monthly)
Repeatedly (every other day-weekly)
Daily
Several times per day

8.2. Describe a typical chewing/licking episode _____________

8.3. How long on average does your dog spend chewing or licking himself/herself during one day? *
0 - 30 min
30 min - 1 hour
1 - 2 hours
2 - 5 hours
5 hours or more

8.4. On average, how long does your dog's one chewing or licking episode last? *
0 - 5 sec
5 - 30 sec
30 sec - 1 min
1 - 5 min
5 - 10 min
10 - 15 min
15 min - 1 hour
More than 1 hour

8.5. Does chewing/licking negatively affect your dog's daily activities or other functions? * (If answer is ″Yes″, Q8.5.1. is shown.)

Yes
No
I don't know

8.5.1. Please specify, how: ______________

8.6. Have you told (ordered/commanded) your dog to stop chewing/licking? * (If answer is "Yes", Q8.6.1. is shown.)
Yes
No
I don't know

8.6.1. Does your dog stop chewing/licking when requested? *
Yes, most of the time
Yes, sometimes
Yes, but soon starts again e.g. in another room
No

8.7. During chewing/licking, does your dog react to his/her name or other commands? *
Yes, like normally
Yes, but worse than normally
Not usually
Not at all

8.8. Does your dog have skin injuries due to biting/licking himself/herself? *
No
Yes, minor injuries (redness of the skin/hairless spots)
Yes, major injuries (wound/skin infection)

8.9. Does chewing/licking occur during a specific situation? You can choose multiple options. *
The behavior does not occur during any specific situation
Excitement/arousal
Playing, play-fighting
Stress, frustration
Going for/coming from a walk
When the dog is bored and has too much energy
Skin itching
The behavior occurs during a specific time of the day
Something else (if ticked, Q8.9.1. is shown)

8.9.1. Please specify: __________

8.10. When did chewing or licking begin? *
Before 3 months of age
At 3 months - 6 months of age
At 6 months - 1 year of age
At 1 year - 1.5 year of age
At 1.5 - 2 years of age
At 2 - 3 years of age
At 3 - 6 years of age
Older than 6 years of age
My dog had stereotypic behavior before I got him/her

9. Flank sucking - Dog sucks on the fur or skin on her/his flank (the area above the thigh) for a long time. Does your dog suck her/his flank? * (If answer is ”More often (at least once a year)”, Q9.1-Q9.9. are shown.)
I've never noticed this behavior
A few times during the dog's lifetime
More often (at least once a year)

9.1. Please specify, how often your dog sucks her/his flank. *
Every once in a while (monthly-yearly)
Quite often (weekly-monthly)
Repeatedly (every other day-weekly)
Several times per day
Daily

9.2. Describe a typical sucking episode ______________

9.3. How long on average does your dog spend flank sucking during one day? *
0 - 30 min
30 min - 1 hour
1 - 2 hours
2 - 5 hours
5 hours or more

9.4. On average, how much time does your dog's one sucking episode last? *
0 - 5 sec
5 - 30 sec
30 sec - 1 min
1 - 5 min
5 - 10 min
10 - 15 min
15 - 1 hour
More than 1 hour

9.5. Does flank sucking negatively affect your dog's daily activities or other functions? * (If answer is ″Yes″, Q9.5.1. is shown.)

Yes
No
I don't know

9.5.1. Please specify: ____________

9.6. Have you told (ordered/commanded) your dog to stop flank sucking? * (If answer is ″Yes″, Q9.6.1. is shown.)

Yes
No
I don't remember

9.6.1. Does your dog stop sucking when requested? *

Yes, most of the time
Yes, sometimes
Yes, but soon stars again e.g. in another room
No

9.7. During flank sucking, does your dog react to his/her name or other commands? *

Yes, like normally
Yes, but worse than normally
Not usually
Not at all

9.8. Does flank sucking occur during a specific situation? You can choose multiple options. *

The behavior does not occur during any specific situation
Stress, frustration
The dog is bored, has too much energy
Discomfort, pain
Being alone
Before going to sleep or rest
The behavior occurs during a specific time of the day
Something else (if ticked, Q9.8.1. is shown)

9.8.1. Please specify: _____________

9.9. When did flank sucking begin? *

Before 3 months of age
At 3 months - 6 months of age

At 6 months - 1 year of age
At 1 year - 1.5 year of age
At 1.5 - 2 years of age
At 2 - 3 years of age
At 3 - 6 years of age
Older than 6 years of age
My dog had stereotypic behavior before I got him/her

10. Eating inedible objects (pica) - Dog eats inedible objects such as socks or rocks. Does your dog eat inedible objects? * (If answer is ”More often (at least once a year)”, Q10.1-Q10.9. are shown.)
I've never noticed this behavior
A few times during the dog's lifetime
More often (at least once a year)

10.1. Please specify how often your dog eats inedible objects. *
Every once in a while (monthly-yearly)
Quite often (weekly-monthly)
Repeatedly (every other day-weekly)
Daily
Several times per day

10.2. Describe a typical pica episode ________________

10.3. How long on average does your dog spend eating inedible objects during one day? *
0 - 30 min
30 min - 1 hour
1 - 2 hours
2 - 5 hours
5 hours or more

10.4. On average, how long does your dog's one pica episode last? *
0 - 5 sec
5 - 30 sec
30 sec - 1 min
1 min - 5 min
5 min - 10 min
10 min - 15 min
15 min - 1 hour
more than 1 hour

10.5. Does pica negatively affect your dog's daily activities or other functions? * (If answer is ”Yes”, Q10.5.1. is shown.)
Yes

No
I don't know

10.5.1. Please specify, how: _____________

10.6. Have you told (ordered/commanded) your dog to stop eating inedible objects? * (if answer is ”Yes”, Q10.6.1. is shown.)
Yes
No
I don't remember

10.6.1. Does your dog stop eating when requested? *
Yes, most of the time
Yes, sometimes
Yes, but soon starts again e.g. in another room
No

10.7. During pica, does your dog react to his/her name or other commands? *
Yes, like normally
Yes, but worse than normally
Not usually
Not at all

10.8. Does pica occur during a specific situation? You can choose multiple options. *
The behavior does not occur during any specific situation
Excitement/arousal
Playing, play-fighting
The dog is bored, has too much energy
The behavior occurs during a specific time of the day
Stress, frustration
Going for/coming back from a walk
Being alone
Arrival of the owner or a quest
Something else (if ticked, Q10.8.1. is shown)

10.8.1. Please specify: __________________

10.9. When did pica begin? *
Before 3 months of age
At 3 months - 6 months of age
At 6 months - 1 year of age
At 1 year - 1.5 year of age

At 1.5 - 2 years of age
At 2 - 3 years of age
At 3 - 6 years of age
Older than 6 years of age
My dog had stereotypic behavior before I got him/her

11. Is your dog given medication for stereotypic behavior? * (if answer is ″Yes″, Q11.1. is shown.)
Yes
No

11.1. Tell the name of the medication ______________

12. Does your dog have some other stereotypic behaviors? ___________________

13. If you want, you can write further details about your dog's behavior. _________________

Personality survey

The questions in this survey consist of an adjective and it's description. From the answering option, choose the option that best fits your dog. Try to answer all the statements and choose "I don't know" only if your dog has not been in the described situation (e.g. your dog has not met other dogs of the same gender). If you answer "I don't know" to a question, the report of that part of the questionnaire will be empty.

Asterisk indicates mandatory field.

Data protection *
Hereby, I accept that my personal information, the information of my dog and all of the data collected with this questionnaire I have provided is transferred to Canine Genetics research group at the University of Helsinki and used in scientific research. Read the privacy policy of the Canine Genetics Group (only in Finnish).

1. Willing to learn: Easy to motivate, likes challenges, gets excited during training sessions. *
    Strongly disagree
    Somewhat disagree
    Neither agree or disagree
    Somewhat agree
    Strongly agree
    I don't know

2. Playful (with dogs): Initiates and engages to play with other dogs. *
    Strongly disagree
    Somewhat disagree
    Neither agree or disagree
    Somewhat agree
    Strongly agree
    I don't know

3. Predictable: Behavior is consistent and steady over extended periods of time. Does little that is unexpected or deviates from its usual behavioral routine. *
    Strongly disagree
    Somewhat disagree
    Neither agree or disagree
    Somewhat agree
    Strongly agree
    I don't know

4. Obedient: Compliant, willingly behaves when asked to do something. *
    Strongly disagree

Somewhat disagree
Neither agree or disagree
Somewhat agree
Strongly agree
I don't know

5. Boisterous: Plays roughly, might start jumping up when greeting someone. *
Strongly disagree
Somewhat disagree
Neither agree or disagree
Somewhat agree
Strongly agree
I don't know

6. Sensitive to touch: Does not like to be touched (e.g. petted), and responds strongly to touching, e.g. by stepping aside or startling. *
Strongly disagree
Somewhat disagree
Neither agree or disagree
Somewhat agree
Strongly agree
I don't know

7. Vocal: Frequently and readily vocalizes. *
Strongly disagree
Somewhat disagree
Neither agree or disagree
Somewhat agree
Strongly agree
I don't know

8. Anxious: Nervous, restless. Is scared easily, 'neurotic' and fearful in general. Does not want to approach scary things or people. *
Strongly disagree
Somewhat disagree
Neither agree or disagree
Somewhat agree
Strongly agree
I don't know

9. Independent: Behavior not influenced or limited by other dogs or people. Does not need constant guidance. *
Strongly disagree

Somewhat disagree
Neither agree or disagree
Somewhat agree
Strongly agree
I don't know

10. Lazy: Disinclined to action or exertion. *
Strongly disagree
Somewhat disagree
Neither agree or disagree
Somewhat agree
Strongly agree
I don't know

11. Intelligent: Learns quickly to associate certain events and appears to remember for a long time. *
Strongly disagree
Somewhat disagree
Neither agree or disagree
Somewhat agree
Strongly agree
I don't know

12. Reliable: Can be trusted in any situation. *
Strongly disagree
Somewhat disagree
Neither agree or disagree
Somewhat agree
Strongly agree
I don't know

13. Prey driven (chase): Is interested in and chases/tries to chase moving objects, for example, toys, other animals or cars. *
Strongly disagree
Somewhat disagree
Neither agree or disagree
Somewhat agree
Strongly agree
I don't know

14. Human-dependent: Readily asks for help from people, instead of trying to figure out the problem independently by herself/himself. *
Strongly disagree

Somewhat disagree
Neither agree or disagree
Somewhat agree
Strongly agree
I don't know

15. Wary: Cautiously watches for possible threats in the surroundings. *
Strongly disagree
Somewhat disagree
Neither agree or disagree
Somewhat agree
Strongly agree
I don't know

16. Erratic: Inconsistent, indefinite, and widely varying in behavior and moods. *
Strongly disagree
Somewhat disagree
Neither agree or disagree
Somewhat agree
Strongly agree
I don't know

17. Stubborn: Persistently does what it wants and continues this behavior despite unfavorable consequences or threats from others. *
Strongly disagree
Somewhat disagree
Neither agree or disagree
Somewhat agree
Strongly agree
I don't know

18. Fearful (of people): Afraid of people; is defensive, hides or escapes. *
Strongly disagree
Somewhat disagree
Neither agree or disagree
Somewhat agree
Strongly agree
I don't know

19. Playful (with people): Initiates and engages to play with people. *
Strongly disagree
Somewhat disagree

Neither agree or disagree
Somewhat agree
Strongly agree
I don't know

20. Playful (alone): Plays a lot and enjoys to play by herself/himself. *
Strongly disagree
Somewhat disagree
Neither agree or disagree
Somewhat agree
Strongly agree
I don't know

21. Curious: Readily explores and is interested in new situations, objects and animals. *
Strongly disagree
Somewhat disagree
Neither agree or disagree
Somewhat agree
Strongly agree
I don't know

22. Fearful (of other dogs): Afraid of other dogs; is defensive, hides or escapes. *
Strongly disagree
Somewhat disagree
Neither agree or disagree
Somewhat agree
Strongly agree
I don't know

23. Impulsive: Often displays spontaneous or sudden behavior that could not have been anticipated. Acts carelessly and hastily. *
Strongly disagree
Somewhat disagree
Neither agree or disagree
Somewhat agree
Strongly agree
I don't know

24. Affectionate (with people): Seeks physical closeness with people. For example, sleeps or relaxes next to people or on their lap, begs petting. *
Strongly disagree
Somewhat disagree

Neither agree or disagree
Somewhat agree
Strongly agree
I don't know

25. Decisive: Purposeful and determined in its activities. *
Strongly disagree
Somewhat disagree
Neither agree or disagree
Somewhat agree
Strongly agree
I don't know

26. Persevering: Tends to continue in a course of action for a long time, does not give up. *
Strongly disagree
Somewhat disagree
Neither agree or disagree
Somewhat agree
Strongly agree
I don't know

27. Distractible: Is easily disturbed by external stimuli (e.g. during training session), cannot concentrate on a task. *
Strongly disagree
Somewhat disagree
Neither agree or disagree
Somewhat agree
Strongly agree
I don't know

28. Submissive: Appeasing or submissive towards other dogs. Easily gives up to others, does not provoke a fight. *
Strongly disagree
Somewhat disagree
Neither agree or disagree
Somewhat agree
Strongly agree
I don't know

29. Cautious: Acts and investigates new things carefully and cautiously. *
Strongly disagree
Somewhat disagree
Neither agree or disagree
Somewhat agree

Strongly agree
I don't know

30. Restless: Unsettled, does not like standing still. Hard to settle down. *
Strongly disagree
Somewhat disagree
Neither agree or disagree
Somewhat agree
Strongly agree
I don't know

31. Confident: Self-assured, certain. Does not hesitate to act, is not scared easily, is not fearful. *
Strongly disagree
Somewhat disagree
Neither agree or disagree
Somewhat agree
Strongly agree
I don't know

32. Easygoing: Does not seem to be worried or anxious of things going on in his/her surroundings. Dog acts, moves and behaves in a relaxed way. *
Strongly disagree
Somewhat disagree
Neither agree or disagree
Somewhat agree
Strongly agree
I don't know

33. Dominant: Is overbearing and/or threatening towards other dogs. *
Strongly disagree
Somewhat disagree
Neither agree or disagree
Somewhat agree
Strongly agree
I don't know

34. Patient: Is not easily agitated or frustrated, is capable of waiting even for a long time. *
Strongly disagree
Somewhat disagree
Neither agree or disagree
Somewhat agree
Strongly agree

I don't know

35. Sociable (with dogs of the same gender): Appears to like the company of other dogs of the same gender. Seeks company of other dogs of the same gender and wants to spend time with them. *

Strongly disagree
Somewhat disagree
Neither agree or disagree
Somewhat agree
Strongly agree
I don't know

36. Slow: Moves slowly and deliberately; not easily hurried. *

Strongly disagree
Somewhat disagree
Neither agree or disagree
Somewhat agree
Strongly agree
I don't know

37. Sociable (with dogs of the opposite gender): Appears to like the company of other dogs of the opposite gender. Seeks company of other dogs of the opposite gender and wants to spend time with them. *

Strongly disagree
Somewhat disagree
Neither agree or disagree
Somewhat agree
Strongly agree
I don't know

38. Calm: Equable, restful. Reacts to others in an even, calm way. Is not easily disturbed or agitated. *

Strongly disagree
Somewhat disagree
Neither agree or disagree
Somewhat agree
Strongly agree
I don't know

39. Provocative: Tests humans or other animals to see what they can get away with. *

Strongly disagree
Somewhat disagree
Neither agree or disagree
Somewhat agree
Strongly agree

I don't know

40. Excitable: Reacts strongly to changes in environment, easily aroused, quickly responds to stimuli. *
Strongly disagree
Somewhat disagree
Neither agree or disagree
Somewhat agree
Strongly agree
I don't know

41. Human-oriented: Enjoys the company of people more than the company of other dogs. *
Strongly disagree
Somewhat disagree
Neither agree or disagree
Somewhat agree
Strongly agree
I don't know

42. Focused: Is focused on what it is doing despite external stimuli. *
Strongly disagree
Somewhat disagree
Neither agree or disagree
Somewhat agree
Strongly agree
I don't know

43. Prey driven (search): Tracks prey animals, starts following scent trails of prey animals. *
Strongly disagree
Somewhat disagree
Neither agree or disagree
Somewhat agree
Strongly agree
I don't know

44. Empathic: Able to understand and read mood of people on the basis of subtle, minimal cues and acts like it's trying to comfort people (e.g comes close, lays his/her head on a person's lap). *
Strongly disagree
Somewhat disagree
Neither agree or disagree
Somewhat agree
Strongly agree
I don't know

45. Aggressive (to dogs of the same gender): Causes harm or threatens to cause harm to other dogs of the same gender, often reacts in a hostile way, defends his/her resources aggressively. For example, the dog can growl or try to snap/bite. *

Strongly disagree
Somewhat disagree
Neither agree or disagree
Somewhat agree
Strongly agree
I don't know

46. Greedy: Eats greedily or in large quantities, has excessive appetite. *

Strongly disagree
Somewhat disagree
Neither agree or disagree
Somewhat agree
Strongly agree
I don't know

47. Attention seeking: Vocalizes to get attention or in another way tries to attract people's attention. *

Strongly disagree
Somewhat disagree
Neither agree or disagree
Somewhat agree
Strongly agree
I don't know

48. Indifferent: Is indifferent towards other dogs, does not seek their company but tolerates them. *

Strongly disagree
Somewhat disagree
Neither agree or disagree
Somewhat agree
Strongly agree
I don't know

49. Aggressive (to people): Causes harm or threatens to cause harm to people, often reacts in a hostile way, defends his/her resources aggressively. For example, the dog can growl or try to snap/bite. *

Strongly disagree
Somewhat disagree
Neither agree or disagree
Somewhat agree
Strongly agree
I don't know

50. Affectionate (with dogs): Seeks physical closeness with familiar dogs. For example, sleeps side by side with other dogs or grooms them. *

Strongly disagree
Somewhat disagree
Neither agree or disagree
Somewhat agree
Strongly agree
I don't know

51. Vigilant: Spends a lot of time attending to his/her surroundings. *

Strongly disagree
Somewhat disagree
Neither agree or disagree
Somewhat agree
Strongly agree
I don't know

52. Calming: Senses the mood of other dogs and is capable of calming them down with calming signals. Dog can, for example, yawn, turn its sight away from the other dog or smell surroundings. *

Strongly disagree
Somewhat disagree
Neither agree or disagree
Somewhat agree
Strongly agree
I don't know

53. Solitary: Wants and prefers to spend time alone, does not seek the company of others. *

Strongly disagree
Somewhat disagree
Neither agree or disagree
Somewhat agree
Strongly agree
I don't know

54. Aggressive (to dogs of the opposite gender): Causes harm or threatens to cause harm to other dogs of the opposite gender, often reacts in a hostile way, defends its resources aggressively. For example, dog can growl or try to snap/bite. *

Strongly disagree
Somewhat disagree
Neither agree or disagree
Somewhat agree
Strongly agree

I don't know

55. Territorial: Is strict with his/her territory; does not allow dogs or people to come to his/her yard/home if the owner is not present. *

Strongly disagree
Somewhat disagree
Neither agree or disagree
Somewhat agree
Strongly agree
I don't know

56. Flexible: Adapts to different situations and is able to modify his/her behavior in them. *

Strongly disagree
Somewhat disagree
Neither agree or disagree
Somewhat agree
Strongly agree
I don't know

57. Easily recovered: After being frightened or excited, dog quickly returns to his/her previous state of mind. *

Strongly disagree
Somewhat disagree
Neither agree or disagree
Somewhat agree
Strongly agree
I don't know

58. Bold: Behaves in a bold, self-confident manner. Is not fearful in new or strange situations. *

Strongly disagree
Somewhat disagree
Neither agree or disagree
Somewhat agree
Strongly agree
I don't know

59. Active: Moves about a lot, spends little time idle. *

Strongly disagree
Somewhat disagree
Neither agree or disagree
Somewhat agree
Strongly agree
I don't know

60. Energetic: Always ready for action, seems to have lot of energy. *

Strongly disagree
Somewhat disagree
Neither agree or disagree
Somewhat agree
Strongly agree
I don't know

61. Insecure: Interested but fearful and uneasy towards new things, vacillates between approach and withdrawal. May need encouragement from people. *

Strongly disagree
Somewhat disagree
Neither agree or disagree
Somewhat agree
Strongly agree
I don't know

62. Attentive: Seems to listen and pay close attention to everything you say or do. *

Strongly disagree
Somewhat disagree
Neither agree or disagree
Somewhat agree
Strongly agree
I don't know

63. Sociable (with people): Appears to like the company of people. Seeks company of people and wants to spend time with them. *

Strongly disagree
Somewhat disagree
Neither agree or disagree
Somewhat agree
Strongly agree
I don't know

64. If you want, you can write further details about your dog's behavior. ______________________________

Breed information (for mixed breed dogs)

Please specify, what known breed your dog has. Start with the breed which your dog has most of for example if your dog has parents that are Spanish Water Dog – Jack Russel Terrier-mix and Spanish Water Dog, fill first breed as Spanish Water Dog and second breed as Jack Russel Terrier. If your dog's parents are different breeds for example Golden Retriever and Finnish Lapponian Dog, you can decide which breed to fill first. Fill as many fields as your dog has breeds. You can leave rest of the fields empty. If your dog has unknown breed, write it on free text field.

1. Breed 1 *

2. Breed 2

3. Breed 3

4. Breed 4

5. Breed 5

6. Breed 6

7. What other breeds does your dog have? You can also write here, if there is some unknown breed.

———————————————

8. Attach here, if you have DNA test results of your dog for example from Embark.
ATTACH FILE

9. How do you know about your dog's breeds? You can choose multiple options. *
    I have guessed breeds based on my dog's look
    The owner of my dog's dam told me
    I have met my dog's dam
    I have met my dog's sire
    I have made DNA test for my dog
